
JOURNAL INFORMATION
==============================
NLM Title Abbreviation: Innov Surg Sci
Journal ID: Innov Surg Sci
NLM Journal ID: 101708165
Journal ID: iss

Innovative Surgical Sciences

EISSN: 2364-7485
Publisher: De Gruyter

ARTICLE INFORMATION
==============================
PMCID: PMC11917182
PMID: 40110106
DOI: 10.1515/iss-2025-2004
Article ID: iss-2025-2004
Article version: 1
Subjects: Article

Abstracts DGKCH

Electronic publication date: 2025 Mar 19
Publication date: 2025 Mar
Volume: 10
Issue: Suppl1
First page: 209
Last page: 255
Copyright: © 2025 by Walter de Gruyter Berlin/Boston
Copyright year: 2025
Copyright holder: De Gruyter
License: This work is licensed under the Creative Commons Attribution 4.0 International License.
License URL: https://creativecommons.org/licenses/by/4.0/

Page count: 47

==============================

**Necrotizing pneumonia in children: Early recognition of indicators for optimal timing of surgical intervention following conservative management – A treatment approach**
ID: 44

**Kategorie: DGKCH - Entzündliche Veränderungen der Lunge**

Ricarda Fischbach[1], Benedikt Wilke[1], Guido Seitz[1], Mohammad Vahdad[1]

[1]*University Hospital Giessen-Marburg, Department of Pediatric Surgery, Marburg*

**Background:**
Necrotizing pneumonia (NP) is a rare, but serious complication of pneumonia, with a rising incidence. NP can lead to prolonged disease and increased mortality, particularly in children. NP is characterized by the destruction of lung tissue and the frequent need for surgical intervention (SI) after unsuccessful conservative treatment. NP is associated with a higher risk of complications, including empyema, pyopneumothorax, bronchopleural fistula (BPF) and sepsis. An early prediction and targeted management are crucial for rapid reconvalescence. Currently, there are no evidence-based recommendations for the diagnosis, treatment and optimal timing of SI for patients with NP. This study aims to identify the potential predictors of complicated pneumonia (CP) in patients who have undergone SI for NP.

**Materials and methods:**
The analysis includes clinical data from all patients conservatively and surgically managed on an interdisciplinary basis for CP and NP between 2017 and 2024. Demographic data (age, sex, diagnosis, antibiotic therapy, pathogen detection, length of hospital stay, surgical procedure, time to surgery post-admission, complications, maximum oxygen demand, type of respiratory support, vital signs inflammation values, fever, comorbidities, allergies, chronic medications, vaccinations, and postoperative management) were retrospectively analyzed, including the results of imaging procedures.

**Results:**
Twelve patients (m/f = 5/7) underwent SI for CP following unsuccessful conservative treatment. The median age was 4 ± 3.2 years. The average hospital stay for the patients with NP was 38 ± 10.3 days. The first SI occurred on average 6 ± 1.27 days post-admission. All patients initially underwent video-assisted thoracoscopic surgery with pleurolysis and chest tube insertion. Three patients previously received chest tubes. Two patients required partial lung resection, one during the primary surgery and the other on the 13th postoperative day. BPF were present in 3/5 NP patients, 2 managed by intervention with occluders. 80% of the NP patients had recurrent fever under antibiotic therapy and increased oxygen demand the day before surgery, whereas all patients with CP remained stable under conservative treatment. The inflammatory parameters could not be identified as indicators for SI. Three patients with NP received high-flow nasal cannula oxygen and one was intubated for bilevel positive airway pressure. All NP patients presented with tachypnea, tachycardia, and ultrasound-confirmed lung consolidation, with 3/4 showing septated pleural effusions. Preoperative CT confirmed NP in 3 patients. None of the patients had relevant comorbidities, allergies, immunodeficiencies or medications. Streptococcus pneumoniae was isolated in all NP cases, with one patient also testing positive for Respiratory-Syncytial-Virus and another for Moraxella catarrhalis. All patients received at least two doses of pneumococcal conjugate vaccines. Patients were treated with antibiotics for an average of 44 ± 5.2 days, which were completed either at discharge or during follow-up. Follow-up checks were carried out between weeks 1-4 after discharge, after 4 months and 6 months - 1 year. 80% of the patients made a full recovery with no pathologies in the chest X-ray and had a normal lung function. The follow-up of one patient has not yet been completed.

**Conclusion:**
The NP treatment in children is challenging and requires interdisciplinary collaboration. Important indicators for early transitioning to SI under escalated antibiotic therapy can be fever, increasing oxygen demand, tachycardia, tachypnea, and radiological evidence of septate effusions, cavitations, and BPF. Early diagnosis by chest ultrasound and CT is crucial for initiating SI. Partial lung resection and fistula closure are often unavoidable in case of unsuccessful conservative treatment. Careful preoperative management and comprehensive postoperative care, including pathogen identification, a long-term antibiotic therapy, and close follow-up, could minimize the risk of late complications. Further research with larger studies is needed to determine the best treatment strategies for children with CP and NP.

Open Access. © 2025 The Author(s), published by De Gruyter. This work is licensed under the Creative Commons Attribution 4.0 International License.

### Are Parapneumonic Effusions and Empyemas Becoming More Complex in the Post-Pandemic Era? A 24-Month Retrospective Analysis of Incidence and Pathogen Spectrum

ID: 578

**Kategorie: DGKCH - Entzündliche Veränderungen der Lunge**

Julia Scholsching[1], Katja Knab[2], Julian Busch[1], Christoph Fusch[2], Karl Bodenschatz[1]

[1]*Klinikum Nürnberg Süd, Klinik für Kinder- und Jugendchirurgie und Kinderurologie, Nürnberg, Deutschland*, [2]*Klinikum Nürnberg Süd, Klinik für Neugeborene, Kinder und Jugendliche, Nürnberg, Deutschland*

**Background:**

Parapneumonic effusions and pleural empyemas are complications of pulmonary infections and often require multidisciplinary treatment. Subjectively, we have observed an increase in the incidence and number of complicated cases in our hospital in the post-pandemic era, which may be due to changes in the spectrum of pathogens. In this retrospective analysis, we present the incidence, clinical courses and treatment outcomes of children with parapneumonic effusions at our institution. The aim is to better understand post-pandemic developments and optimizing therapeutic approaches.

**Materials and methods:**

We conducted a retrospective analysis of all pediatric patients within the last 24 months who were diagnosed with parapneumonic effusion in our pediatric department. Analysis included detected pathogens, antibiotic treatment and the incidence of pleural drainaging and video-assisted thoracoscopic surgery (VATS).

**Results:**

Within 24 months 17 children with parapneumonic effusions were treated in our hospital, aged 10 months to 14 years. 23.5% received an oral antibiotic therapy prior to admission. In 94% of cases the effusion was unilateral. Ten children needed a pleural drainage (58.8%). Median duration of drainage in patents without subsequent VATS was 3 days. Of the 10 children who required pleural drainage 5 (50%) underwent VATS. Major indications for VATS were persistent mediastinal shift, sepsis, deterioration of oxygenation and / or failure of improvement under antibiotic therapy and drainage. Pathogens were found in 88.4% of all patients, 76.5% were detected via PCR.

In 7 out of 10 children with pleural drainage bacterial detection was achieved either via cultures or PCR. Main pathogens found in drainage aspirates were Streptococcus pneumoniae (28.6%) and Streptococcus pyogenes (28.6%). Haemophilus influenzae, Streptococcus intermedius and Mycobacterium tuberculosis were detected in one individual case each. The patient with Streptococcus intermedius developed a complicated and prolonged clinical course. In children without drainage 4 (57.4%) out of 7 were tested positive for Streptococcus pneumoniae in oropharyngeal swabs. All except for two patients were treated with Ampicillin/ sulbactam or Piperacillin/ tazobactam. 35.3% additionally received macrolide antibiotics.

**Conclusion:**

Similar to the initial reports from other hospitals, our clinic has also seen a significant increase in pleural empyemas requiring intervention since 2023. In the previous period from 2021-2023, we performed no VATS for pleural empyemas. However, unlike previous publications, our case series did not show a change in the pathogen spectrum. Larger case studies are needed to evaluate additional underlying epidemiological factors.

**Suitability of Appendix Tip Biopsy as a Diagnostic Tool for Total Colonic Aganglionosis**
ID: 325

**Kategorie: DGKCH - Herausforderungen bei komplexen urogenitalen und anorektalen Fehlbildungen**

Laura Stigler[1], Carla Sternke- Curvello[1], Andreas Erbersdobler[2], Stefanie Märzheuser[1], Judith Lindert[1]

[1]*Klinik für Kinderchirurgie , Rostock , Deutschland*, [2]*Institut für Pathologie , Rostock , Deutschland*

**Background:**
Total Colonic Aganglionosis (TCA) is a rare variant of Hirschsprung's disease (HD) with an incidence of about 1:50,000-100,000 live births.
The time to establish the diagnosis depends on the clinical presentation but mainly on the awareness of medical staff towards this rare disease. Often children undergo multiple surgeries before patients receive the correct diagnosis and corresponding therapy.
Some studies have already tried to show whether an appendix tip biopsy for ganglia cell (GC) detection is suitable for confirming TCA with the potential to establish a diagnosis.

**Materials and methods:**
Within the framework of a prospective, exploratory, non-randomized longitudinal study, all patients up to the age of 15 years were included who underwent abdominal surgery with an appendix tip biopsy between 10/2022 and 09/2024.
The patients were divided into three cohorts: Cohort A (HD), Cohort B (TCA) and Cohort C (Non-HD, Non-TCA).

**Results:**
Cohort A included 24 patients, 17 male and 7 female with a mean age of 20.1 months (4-156) at the time of the Appendix biopsy.
Cohort B included 26 patients, 20 male and 6 female with a mean age of 43.6 months (3-132) at the time of the Appendix biopsy.
Cohort C included 51 patients, 33 male and 18 female with a mean age of 7.7 years (0-15) at the time of the Appendix biopsy. The indications for surgery in this cohort were 75% acute appendicitis, 7.8% recurrent Appendicitis after conservative treatment, and 17.6% other indications.
In Cohort A, ganglia cells were detectable in the appendix biopsy in 95.8% (N=23). Only in one patient, no GCs were detectable, attributable to an insufficient biopsy (only mucosal biopsy). Overall, GCs could be detected with a sensitivity of 95.8% in this cohort.
Cohort B included 10 patients who no longer had an appendix due to various previous surgeries. In the remaining 16 patients, no GCs were detectable in 93.7%. In one patient, a GC was detected in the appendix biopsy.
In Cohort C, GCs were detectable in the appendix biopsy in 96.1% (N=51). In two patients, reliable detection was not possible due to advanced inflammatory processes. In this cohort, GCs could be detected with a sensitivity of 96.1%.
Overall, the absence of GCs could be detected in all cohorts with a sensitivity of 93.8% and a specificity of 96%.
The positive predictive value for the absence of GCs is 83.3%. The negative predictive value is 98.6%.

**Conclusion:**
When performed correctly, the appendix tip biopsy can be used as a reliable diagnostic tool for the detection or absence of ganglion cells.
There were no differences between newborns and older children regarding the possibility of detecting ganglion cells.
An advanced inflammatory process can be a limiting factor for ganglion cell detection due to massive tissue destruction.
Summarizing a biopsy of the appendix is a potential diagnostic tool to diagnose TCA.

**Reducing the postoperative rate of Hirschsprung associated enterocolitis: a retrospective study**
ID: 333

**Kategorie: DGKCH - Herausforderungen bei komplexen urogenitalen und anorektalen Fehlbildungen**

Hannah Luz[1], Philipp Szavay[1], Peter Esslinger[1]

[1]*Kinderchirurgie, Kinderspital Zentralschweiz, Luzern, Schweiz*

**Background:**
Hirschsprung associated enterocolitis (HAEC) is one of the most severe known complications of Hirschsprung’s disease, both pre- and postoperatively. This study explores the efficacy of consequent postoperative bougienage and consequent long-time follow ups in reducing HAEC rates.

**Materials and methods:**
A retrospective analysis encompassing 53 patients who underwent transanal pull through (with and without laparoscopic mobilization) operations between 1999 and 2023 in our institution was conducted. Rigorous follow-up protocols were implemented, incorporating regular bougienage sessions during the initial three months postoperatively. Patient records, including clinical notes and diagnostic tests, were meticulously reviewed to assess the incidence of HAEC.

**Results:**
Among our cohort, out of the 53 children, only one confirmed case of postoperative HAEC was documented, alongside a singular instance of suspected HAEC. This remarkably low HAEC rate of 4% in postoperative cases highlights the potential benefits of our implemented bougienage strategy and consequent regular follow ups with early detection of stenosis and constipation as well as its treatment in preventing HAEC following transanal pull through operations.

**Conclusion:**
Consequent postoperative bougienage, integrated with a meticulous follow up regimen, emerges as a promising approach to mitigate the risk of Hirschsprung Associated Enterocolitis after transanal pull through procedures. These findings suggest that a proactive approach involving regular bougienage and regular follow ups play a pivotal role in preventing the onset of HAEC, a serious complication associated with Hirschsprung's disease.

**Assessing fecal load with point of care ultrasound in children with colorectal pathology: ReKiSo study**
ID: 480

**Kategorie: DGKCH - Herausforderungen bei komplexen urogenitalen und anorektalen Fehlbildungen**

Daniel Erkel[1] [1], Stefanie Märzheuser[1], Judith Lindert[1]

[1]*Universitätsmedizin Rostock, Kinderchirurgie, Rostock, Deutschland*

**Background:**
Children with rare congenital colorectal disorders like Anorectal malformation (ARM) or Hirschsprung disease (HD) often suffer from problems of defecation, constipation or stool soiling after surgical treatment. Thus, bowel management (BM) focuses on achieving social cleanness and continence. BM programs monitor fecal load and their outcome with repetitive abdominal X-rays. Long-term effects of repetitive exposure in children to radiation are described and the relevant age group of < 10 years shows the highest risk of any kind of cancer. For diagnosis or evaluation of fecal load abdominal X-rays are commonly used in children with functional constipation (FC), although utility is considered low, lacks medical evidence and is not recommended in international guidelines. This should be considered because patients with ARM or HD show more readmissions due to constipation and need prolonged BM. Literature research presents abdominal ultrasound examination in children with FC as an alternative tool equivalent to abdominal X-rays. So far patients with ARM or HD are consistently excluded in these studies and there is no data. In consequence, the aim of this study is to evaluate the point of care ultrasound evaluation of the transverse rectal diameter (TRD) to assess fecal and monitor bowel management in children with colorectal pathology.

**Materials and methods:**
Prospective case-control study of children attending the colorectal outpatient clinic between 04/2023 and 04/2024. Inclusion of patients with HD, ARM and FC. Control group consisted of inpatients and outpatients without abdominal complaints. FC was diagnosed according to ROM-IV criteria. For children with HD and ARM we followed a symptom list regarding constipation and fecal incontinence. To assess the fecal load, we visualised the TRD using the Klijn method by placing a curved array transducer over the symphysis and measuring the TRD at a downward angle of at least 15 degrees from the transverse plane. The bladder was moderately full. The fecal load was assessed retrograde from the rectum. Follow-up was at 1/3/6 months. Secondary data (age, weight, height, comorbidity, surgery) were collected from medical records. Sample size calculated a priori with effect size $d = 0.8$ and $\alpha = 0.05$ for case and control groups. Analysis with SPSS version 26.0.

**Results:**
Pooled results are summarized. For the calculation in the HD and ARM groups, we considered patients after pull-through surgery or Posterior/Anterior Sagittal Anorectoplasty (PSARP/ASARP) and excluded those with total aganglionosis (TCA). There was insufficient data for patients younger than one year. We found significantly increased TRD for patients with ARM or HD due to constipation, fecal soiling and associated symptoms. These findings were similar to the data of the literature research on children with FC. P value for TRD in all groups significant with $p < 0.05$ and also during the grouped follow-up. The TRD exceeded the cut-offs comparable to FC and was significantly lowered with successful BM. The collective sensitivity of 95.89% and specificity of 72.84 were equivalent to reported value for FC.
All patients of the control group showed no fecal load in the ampulla recti. Although patients in the case groups reported any symptoms, there was stool present in other parts of the colon ranging from 8.82 to 18.75%. Fecal load increased with the presentation of symptoms from 71.43 to 91.43 %. The correlation between TRD and fecal load was high and statistically significant ($p < 0.0001$). In contrast to the evaluation of fecal load in BM with X-rays this criterion did not influence our concept of BM and the measurement of TRD was already sufficient.

**Conclusion:**
Point of Care ultrasound is a useful tool to assess the fecal load and helps to diagnose constipation and monitor BM in children with and without colorectal pathology. We present new data for children with ARM or HD. Irrespective of bowel pathology, a cut-off of 3 cm seems to discriminate between children without constipation/overload symptoms and those who are doing well. We also present a radiation-free method for monitoring bowel management.

### Penile tourniquet duration is not prolonged in postoperative complicative cases following paediatric hypospadias repair

ID: 538

**Kategorie: DGKCH - Herausforderungen bei komplexen urogenitalen und anorektalen Fehlbildungen**

Anna Messling[1], Malin Dönig[2], Volker Sander[1], Ralf-Bodo Tröbs[3], Matthias Nissen[1]

[1]*Kindermedizinisches Zentrum der St. Elisabeth Gruppe - Klinik für Kinderchirurgie, Witten, Deutschland*, [2]*Gemeinschaftskrankenhaus Herdecke gGmbH, Herdecke, Deutschland*, [3]*Krankenhaus Barmherzige Brüder Regensburg Klinik fkinderchirurgie, Regensburg, Deutschland*

**Background:**
Knowledge on the safety and efficacy of penile tourniquet use during paediatric hypospadias repair is scarce. Experimental data underlines that tourniquet use might pose a risk factor for the development of postoperative complications such as urethral fistula and meatal stenosis. In this study, we focussed on the ischemia duration and its impact on postoperative complicative course in paediatric hypospadias repair.

**Materials and methods:**
A retrospective monocentric review was conducted on children that underwent hypospadias repair under penile tourniquet application for haemostasis during a ten years period. The primary objective was the tourniquet time and its association with postoperative complicative course, defined as the necessity for redo surgery for meatal stenosis or urethral fistula. The secondary objective included biometric and procedural characteristics.

**Results:**
From 438 patients that underwent primary hypospadias repair during a 10 years study period, 112 patients were included with documented ischemia time. Postoperative complicative course was observed in 24 (21%) cases. Comparing complicative with non-complicative postoperative course, penile tourniquet time was not different among groups (37 (32-45) vs. 35 (30-45) min; p= 0.78), nor was the patient age (15.5 (10-30) vs. 16 (11-30) months; p= 0.85), weight (12 (10-17) vs. 12 (10-15) kg; p= 0.51), and follow up time (10 (8-11) vs. 9 (8-10) years; p= 0.20).

**Conclusion:**
Tourniquet time has not previously been investigated as a primary factor impacting on postoperative development of meatal stenosis or urethral fistula in paediatric hypospadias repair. In this study, we did not observe any differences regarding penile tourniquet time among groups. Hence, tourniquet time may not be a primary factor for the development of a complicative postoperative course. Further research is mandatory to define the role of tissue ischemia in the development of complications after paediatric hypospadias repair.

**Proximal hypospadias – navigating complex urogenital anomalies in a multidisciplinary setting**
ID: 634

**Kategorie: DGKCH - Herausforderungen bei komplexen urogenitalen und anorektalen Fehlbildungen**

Christina Campbell[1], Ilmi Alhussami[1]

[1]*Uniklinikum Jena, Klinik für Kinderchirurgie, Jena, Deutschland*

**Background:**
Technical-surgical management of proximal hypospadias has remained a subject of controversy over the past decades. While some aspects of high quality surgical care have become apparent (Yu et al 2022), postoperative rates for urethrocutaneous fistula and other complications continue to be high. We present our experience of comprehensive management of proximal hypospadias.

**Results:**
Trying better to understand the etiology of many Disorders of Sexual Differentiation, more and more genetical factors have surfaced. Previously, etiology has remained obscure for several patients with proximal hypospadias, even those associated with other urogenital anomalies (such as cryptorchidism, testicular dysplasia, and persisting muellerian structures). However, the likelihood of associated anomalies (including acquired undescended testes) does increase with more proximal forms as does the likelihood of finding chromosomal aberration or other traditional DSD diagnosis – both once again seconded by our data.
Associated anomalies can make further intervention necessary: primary meatal stenosis may cause upper urinary tract anomaly, gonadal variation may necessitate biopsy or pexy, muellerian derivatives may cause infection, functional problems of micturition, and present a risk of malignancy, hypoandrogenism, where present, may be associated with a higher risk of (among others) cardiovascular disease later in life.
Data revealing similar surgical outcomes for patients with proximal hypospadias “with or without DSD diagnosis” cannot accurately compare entities as many underlying factors have yet to be discovered. Additionally, each patient presents with a highly individual composition of anatomical, endocrinological, genetical, and especially psychosocial factors. Because of this, prognosis regarding long-term outcomes remains a challenge.

**Conclusion:**
Current legal standards for surgical intervention in children with DSDs in Germany require an expert report from a multidisciplinary commission, consisting of, for example, pediatric endocrinology, psychology, ethics, and surgery. Our center has taken this albeit controversial requirement as an opportunity to offer patients with different DSD diagnoses and their families enhanced medical, surgical, and psychological care. This includes regular multidisciplinary meetings and early psychosocial assessment of patients, for example, those with proximal hypospadias, and their families. As the etiology remains unknown in several patients with proximal hypospadias, we highly recommend care for these patients in the above-mentioned multidisciplinary setting.

**Abstract: Pentalogy of Cantrell – a prenatal and surgical challenge with a promising outcome. A report of 4 cases.**
ID: 768

**Kategorie: DGKCH - Herausforderungen bei komplexen urogenitalen und anorektalen Fehlbildungen**

Astrid Lackinger[1], Sophie Langer[2], Renate Fartacek[2], Martin Metzelder[2], Wilfried Krois[2]

[1]*Medizinische Universität Wien, Kinderchirurgie, Wien, Österreich*, [2]*Medizinische Universität Wien, Wien, Österreich*

**Background:**
Pentalogy of Cantrell (POC) is a rare congenital disorder characterized by a spectrum of malformations, including anterior abdominal wall defects, sternal cleft, cardiac anomalies, and diaphragmatic hernia. It is associated with high morbidity and mortality, yet timely surgical intervention can significantly improve quality of life.

**Materials and methods:**
**Objective:** This report presents three cases of patients diagnosed with POC and a fourth case with a sternal cleft and cardiac anomalies. It emphasizes the shared challenges of these conditions and the potential for favorable outcomes through surgical management.

**Results:**
**Case Presentations:**
**Case 1:** A newborn female presented with a prenatally diagnosed omphalocele and cardiac defect. Postnatally we found additionally a diaphragmatic defect, sternal defect, tetralogy of Fallot, and a misplaced auricle. Notably, the right auricle of the heart was elongated and adherent to the abdominal wall. Following delivery, diaphragmatic closure was performed using pleura and pericardium, with subsequent staged abdominal wall closure achieved using a Gore-Tex patch. The patient is currently awaiting cardiac repair.
**Case 2**: Another newborn girl was diagnosed prenatally with an omphalocele and suspected POC, including tetralogy of Fallot, retrosternal diaphragmatic defect, pericardial defect and lung hypoplasia. Surgical treatment involved retrosternal diaphragmatic closure, abdominal wall repair, and correction of the cardiac defects. She is now 8 years old, thriving, and developmentally on track.
**Case 3**: A female infant was prenatally diagnosed with omphalocele and delivered via cesarean section due to developing pleural effusions at 35 weeks of gestation. Postpartum evaluation revealed POC with classical findings. Initial surgeries successfully closed the diaphragm and abdominal wall; the pericardial defect was left open. At the age of 2 months she developed colonic necrosis, which resulted in a subtotal colectomie with coecoanal anastomosis.
**Case 4:** A newborn girl diagnosed with a superior congenital sternal cleft presented with scar-like tissue in the middle of her chest and a small omphalocele sac containing the right auricule of the heart in the cranial region of the sternal cleft, suggesting an incomplete POC. Surgical resection of the omphalocele sack and reconstruction of the pericardial defect resulted in a favorable cosmetic and functional outcome.

**Conclusion:**
Cases 1 to 3 demonstrate the typical manifestations of POC along with various treatment options and outcomes. In contrast, Case 4 presents a distinct scenario where the patient exhibited all the clinical findings typically associated with POC; however, instead of the commonly seen omphalocele, there was only a small sac containing the auricle of the heart. This highlights that while the presentation may vary, it still represents the same underlying anomaly and that further research into pathophysiology is necessary. Additionally, early prenatal diagnoses are crucial for guiding clinical management and providing appropriate counseling to parents.

### A Comparative in Vivo Study of Hyperthermic Intraperitoneal Chemotherapy with Cisplatin versus Doxorubicin versus Cisplatin plus Doxorubicin for the Treatment of Intraabdominally Disseminated Alveolar Rhabdomyosarcoma in Mice

ID: 96

**Kategorie: DGKCH - Kinderchirurgische Forschung**

Illya Martynov[1], Jens Gesche[1], Luca Tobi[2], Paul Hoyer[1], Guido Seitz[1]

[1]*Department of Pediatric Surgery and Urology, University Hospital Giessen-Marburg, Baldingerstraße, 35043 Marburg, Germany, Marburg , Deutschland*, [2]*Department of Pediatric Surgery, University Hospital Giessen-Marburg, Rudolf-Buchheim-Strasse 7, 35392 Giessen, Germany, Gießen, Deutschland*

**Background:**

Treatment options for advanced intraabdominal pediatric rhabdomyosarcoma (RMS) with peritoneal sarcomatosis (PS) include cytoreductive surgery (CRS) and hyperthermic intraperitoneal chemotherapy (HIPEC). However, optimal dosages and combination regimens of drugs used for HIPEC are underexplored. We aimed to evaluate the efficacy of HIPEC with cisplatin, doxorubicin, and their combination in vivo.

**Materials and methods:**

We established PS/RMS mouse model by intraperitoneally injecting RH30 cells into NOD/LtSz-scid IL2Rγnull mice. Two weeks post-xenotransplantation, mice underwent a single HIPEC procedure at 42°C for 60 minutes. Treatment groups received cisplatin (50, 100, and 150 mg/m2) and doxorubicin (30, 45, and 60 mg/m2), administered alone or combined. The control group underwent an intraperitoneal lavage with isotonic saline. Peritoneal carcinosis index (PCI) was used to quantify the extent of peritoneal tumor spread. Tissue samples were evaluated regarding proliferation (Ki-67) and apoptosis (Caspase 3).

**Results:**

Mice treated with cisplatin at 100 mg/m2 (PCI of 3.875, $p = 0.007$), 150 mg/m2 (PCI of 4.556, $p = 0.026$), doxorubicin at 30 mg/m2 (PCI of 2.875, $p < 0.001$), and 45 mg/m2 (PCI of 4.143, $p = 0.021$) showed reduced PCI, with the combination of cisplatin 50 mg/m2 and doxorubicin 30 mg/m2 showing the most prominent effect (PCI of 3.333, $p < 0.001$) compared to the control group (PCI of 8.615). Histologically, there was no difference in Ki-67 or Caspase 3 expression among the groups.

**Conclusion:**

The cisplatin- and doxorubicin-based HIPEC significantly reduces peritoneal tumor dissemination in vivo. Further investigations are needed to explore the underlying molecular responses to optimize therapeutic strategies.

**Intussusception in pediatric patients with feeding jejunostomy: Case series and management strategies**
ID: 182

**Kategorie: DGKCH - Kinderchirurgische Forschung**

Nariman Mokhaberi[1], Omid Madadi-Sanjani[1], Johannes Görges[2], Konrad Reinshagen[1], Christian Tomuschat[1]

[1]*Universitätsklinikum Hamburg-Eppendorf, Klinik und Poliklinik für Kinderchirurgie, Hamburg, Deutschland*, [2]*Universitätsklinikum Hamburg-Eppendorf, Klinik und Poliklinik für Diagnostische und Interventionelle Radiologie und Nuklearmedizin, Hamburg, Deutschland*

**Background:**
Jejunal feeding is an established option for providing nutrition to paediatric patients with impaired oral or gastric feeding, severe reflux or gastric outlet obstruction. The jejunal feeding tube (JFT) can be placed endoscopically or surgically. Mechanical complications include small bowel intussusception, which must be distinguished from spontaneous intussusception. Given the limited data available, the aim of our study was to present the cases at our institution and to review the literature to identify potential management approaches.

**Materials and methods:**
A retrospective chart review between the years 2014 and 2024 at the University Medical Center Hamburg-Eppendorf was conducted. Data collection included demographic and chronological data, underlying pathology, type of tube-placement, symptoms at presentation and therapy of the intussusception.

**Results:**
A total of six patients with small bowel intussusceptions around a JTF were identified. The mean age at the time of JT placement was 3.62 years. The mean age at which intussusception occurred was 6.9 years, with a mean duration between placement and intussusception of 3.28 years.
In four patients, the JT was inserted endoscopically; one JT was inserted radiologically and the other was inserted surgically via Witzel's technique. Four JTFs were inserted endoscopically, one radiologically and one surgically. In four patients, a spontaneous reduction was observed, one intussusception was reduced hydrostatically and in one intussusception, the probe was retracted endoscopically.
The literature on intussusception in children with jejunal tubes is limited and mainly consists of case reports and a few retrospective studies. The further course was not discussed in many cases, and the described cases and their management were heterogeneous. Intussusceptions were observed with all different insertion methods, and the therapies included observation, conservative measures and surgical intervention. The existing literature on intussusceptions in adult patients with a jejunal feeding tube is also very limited. Surgical intervention was frequently performed in the cases described, with only a few patients experiencing reduction of the intussusception through conservative treatment measures.

**Conclusion:**
Intussusception is a rare complication in pediatric patients with JTF that can lead to bowel ischemia. It occurs independently of the insertion technique. Patients typically present with abdominal pain, bilious vomiting, and feeding intolerance. Radiological diagnostics include sonography and, if indicated, x-ray and cross-sectional imaging. Our findings align with those of previous studies and suggest a predominantly conservative approach, with the potential for interventional intervention when necessary. In instances of treatment failure or in patients with critical illness, surgical revision of the intussusception is recommended. These results diverge from those observed in cases involving adult patients, where surgical procedures were typically performed.

**Presence of ganglion cells in the appendix from neonates to the elderly – a way to reliable diagnostic of Total Colonic Aganglionosis**
ID: 291

**Kategorie: DGKCH - Kinderchirurgische Forschung**

Carla Antonia Sternke Curvello[1], Laura Stigler[1], Andreas Erbersdobler[2], Redia Hussein[3], Judith Lindert[1]

[1]*Universitätsmedizin Rostock, Klinik für Kinderchirurgie, Rostock, Deutschland*, [2]*Universitätsmedizin Rostock, Institut für Pathologie, Roctock, Deutschland*, [3]*Universitätsmedizin Rostock, Klinik und Poliklinik für Allgemein-, Viszeral-, Thorax-, Gefäß- und Transplantationschirurgie, Rostock, Deutschland*

**Background:**
The identification of absent ganglion cells in the intestinal tract is a crucial factor in diagnosing Hirschsprung´s disease (HD). In cases of total colonic aganglionosis (TCA), examining ganglion cells in the vermiform appendix has been proposed as a potential diagnostic marker. However, the available data on the presence and detectability of ganglion cells in the appendix – both in healthy and pathological tissue samples – is limited and inconsistent. To assess whether the examination of ganglion cells in the appendix has the potential to enhance diagnostic accuracy and improve clinical management, particularly in diagnosing total colonic aganglionosis, we aimed to evaluate the presence of ganglion cells across different age groups and explore the impact of appendicitis and other underlying conditions on their visualization.

**Materials and methods:**
This prospective cohort study involved histopathological examination of the appendix from n=189 pediatric and general surgery patients, ranging in age from 1 day to 89 years. These patients underwent procedures that included resection of the appendix in both pediatric and adult visceral surgery at a single institution from October 2022 to September 2024. Tissue samples were analyzed for the presence of enteric nervous system ganglion cells by a pathologist.

**Results:**
During the study period, a total of 189 patients aged 1 day to 89 years underwent surgery involving the resection of the vermiform appendix. The cohort consisted of 61.38% males and 38.62% females, with a mean age of 31.47 years; 48.68% of patients were under 16 years old. Indications for surgery included appendicitis (n=120), colorectal carcinoma (n=12), Crohn´s disease (n=3), Hirschsprung´s disease (n=41), including 17 cases of total colonic aganglionosis (TCA), and various other conditions (n=13). Histopathological examination revealed present ganglion cells in 97.1% (167/172) of the appendices in the non-TCA-cohort. Only four specimens were unassessable due to severe inflammation, and one appendix from a child with Hirschsprung´s disease lacked ganglion cells. In the TCA-cohort, 94.1% (16 out of 17) of the examined tissue samples were devoid of ganglion cells.

**Conclusion:**
The findings of this study suggest that the presence of ganglion cells in the vermiform appendix is highly consistent in non-TCA cases, including children with common Hirschsprung´s disease. The absence of ganglion cells in TCA cases supports the use of the appendix as a reliable diagnostic marker for TCA. However, severe inflammation may hinder accurate assessment, highlighting the need for careful interpretation in such instances. These results underscore the potential utility of appendix examination in enhancing diagnostic accuracy for TCA, although further research is necessary to establish standardized guidelines.

**Remote consequences of pyloromyotomy in dependence of approach type**
ID: 314

**Kategorie: DGKCH - Kinderchirurgische Forschung**

Andriy Pereyaslov[1], Bohdan Malovanyy[2], Roman Stenyk[2], Ihor Hulavskyi[2]

[1]*National Medical University, Pediatric Surgery, L'viv, Ukraine*, [2]*Clinical Centre of children's healthcare, L'viv, Ukraine*

**Background:**
Hypertrophic pyloric stenosis (HPS) one of the most common causes of vomiting in newborns that required the surgery. While intraoperative complications and immediate results of pyloromyotomy widely presented at literature, the remote consequences, especially in dependence of the method of surgical correction, are poorly studied and have conflicting results.
The aim of this study was to analyze the remote consequences of pyloromyotomy in dependence of type of approach – open (OPMT) or mini-invasive (laparoscopic pyloromyotomy – LPMT).

**Materials and methods:**
Initial evaluation of remote consequences was based on the analyzing answers to questions of «Questionnaire for the pediatric functional gastro-intestinal disorders», which were mailed to 258 patients, which were operated at the period from 1995 till 2024 years, and their parents; the answers were received from 186 (72.1%) respondents, which were included in this study. The re-hospitalization required 62 (33.3%) children, in which roentgenological investigation, endoscopy, and ultrasonography was applied.

**Results:**
Among all respondents, OPMT was performed in 132 (78.1%) and LPMT – in 37 (21.9%) of them. One hundred two (60.4%) children, that included 82 (60.7%) children after OPMT and 30 (58.8%) – after LPMT (p=0.8129), had no complaints.
The main complaint in children after pyloromyotomy, which required re-hospitalization, was the presence of chronic abdominal pain, which was indicated by 40 (21.5%) respondents – in 37 (27.3%) after OPMT and in 3 (5.9%) after LPMT (p=0.0013). The functional gastro-intestinal disorders were noted in 15 (8.9%) children: it was 12 (8.9%) children after OPMT and 3 (5.9%) – after LPMT (p=0.5044). Delayed barium passage on intestine that associated with periodical pain syndrome, pointed on chronic intestinal adhesive disease in 26 (13.98%) of children that mainly revealed after OPMT (17.8% vs. 3.9% p=0.0149). However, the frequency of acute intestinal obstruction among these patients had no significant difference (p=0.3362)
According to the results of endoscopy, the gastro-esophageal (11.8% vs. 3.9%, p=0.1038) and duodeno-gastral reflux more often was after the OPMT (11.1% vs. 3.9%, p=0.1304).

**Conclusion:**
The re-hospitalization after pyloromyotomy more often required patients, in which open approach was applied. By that, the frequency of the gastro-esophageal or duodeno-gastral reflux and chronic intestinal adhesive disease had no correlation with the type of approach.

### Gutsy Pursuit: Confronting the Challenges of Human Enteric Nervous System Cultivation to Analyze its Proinflammatory Capacity in Hirschsprung‘s Disease

ID: 315

**Kategorie: DGKCH - Kinderchirurgische Forschung**

Lucie Rohwäder[1], Pauline Schuppert[1 2], Laia Pagerols Raluy[1], Johanna Hagens[1], Anna Romanova[1], Hans Christian Schmidt[1], Zhongwen Li[1], Magdalena Trochimiuk[1], Konrad Reinshagen[1], Christian Tomuschat[1]

[1]*University Medical Center Hamburg-Eppendorf, Department of Pediatric Surgery, Hamburg, Deutschland*, [2]*Martin Luther University Halle-Wittenberg, Halle-Wittenberg, Deutschland*

**Background:**

Hirschsprung-associated enterocolitis (HAEC) is a serious complication in Hirschsprung’s disease (HSCR), with its underlying mechanisms still not fully understood. The enteric nervous system might play a key role in the pathophysiology of HAEC, given that HSCR involves a congenital lack of functional neural cells in parts of the colon. We previously isolated myenteric plexus from pediatric gut samples and cultivated enteric neurospheres, a three-dimensional cell culture model consisting of aggregated neural crest-derived cells, to study the proinflammatory cytokine release. Preliminary data indicates an altered profile in neurospheres derived from patients with HSCR (unpublished data). However, isolated cell studies may not capture the complex interactions within the intestine. A study by Stavely et al. revealed that the cultivation of murine enterospheres, consisting of all major cell populations present in the tunica muscularis, prior to selection of neural cells, allowed for a significantly higher yield of neural cells for secondary neurosphere culture [1]. Culturing enterospheres might therefore not only be a sufficient way to study the proinflammatory cytokine release in a co-culture of neural and mesenchymal cells, but also address the issue of limited sample availability frequently impeding primary human neurosphere culture. We aim to adapt the culture of enterosphere-derived neurospheres to pediatric gut samples to perform a comparative analysis of the proinflammatory cytokine release between HSCR and controls.

**Materials and methods:**

After isolating the sample via microdissection, we enzymatically dissociated the tunica muscularis and cultured it for up to 14 days to form enterospheres ($n$=26). To enhance enterosphere yield and growth, we explored longer enzyme incubation times, employed fresh rather than frozen samples and tested culture plates with untreated surfaces as well as those treated for low-attachment, adherent, and suspension culture. Enterospheres were characterized through immunofluorescence staining with the neural progenitor marker p75, neuronal markers Tuj1 and PGP9.5 and mesenchymal markers a-SMA and Vimentin. For secondary neurospheres, we dissociated the enterospheres with four different enzyme cocktails ($n$=9). We conducted preliminary experiments with magnetic-activated cell sorting (MACS) using PE-Frizzled-4 antibodies to obtain a purified culture of neural progenitor cells for secondary neurosphere culture ($n$=1).

**Results:**
We successfully generated enterospheres from colon samples of HSCR and control patients, as well as from samples of the ileum and jejunum, in 76.9% of our culture attempts. Higher yields of viable cells for enterosphere culture were achieved using fresh samples and more consistent sphere forming was observed with low attachment plates. Although it was possible to partially dissociate enterospheres, the core spheroid structure persisted even after enzymatic incubation for up to 60 minutes, while the resulting single cells appeared to be over-digested. After filtration, the single-cell suspension was examined under a microscope, revealing a significant reduction in viable cells compared to the initial number of enterospheres used for dissociation. In the MACS attempt, we obtained Frizzled-4-positive and negative cells, yet all cells died within three days.

**Conclusion:**
Our findings emphasize the difficulties associated with culturing human enteric neural stem cell derivatives from primary tissue, while confirming the feasability of established enterosphere models to study HSCR and HAEC. As a result, we are focusing on the enterosphere culture for an initial characterization of their proinflammatory capacity. Released cytokines will be measured in stressed cultured enterospheres from HSCR and control samples to gain insight into how human tunica muscularis cells might be involved in the pathophysiology behind HAEC.

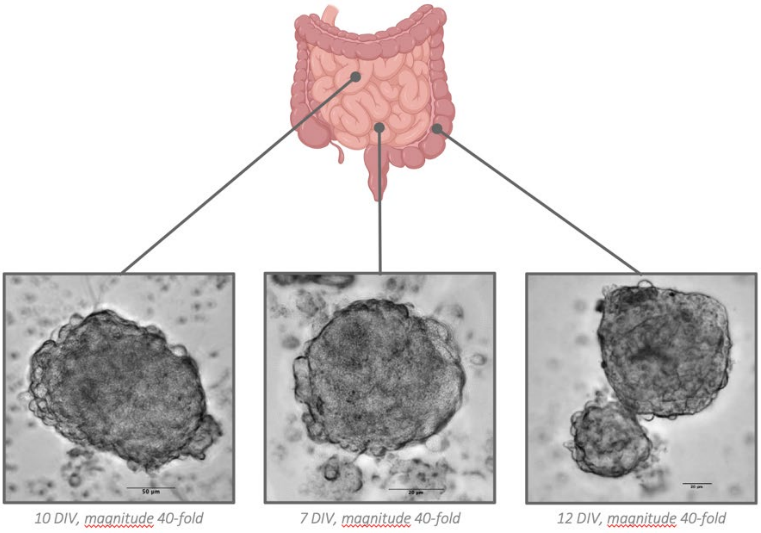

Figure 1: Brightfield images of enterospheres derived from jejunum 10 DIV, ileum 7 DIV, colon 12 DIV (left to right), magnitude 40-fold. Created with Biorender.com.

**Reference:**
[1] Stavely, R, Bhave, S, Ho, WLN, et al. Enteric mesenchymal cells support the growth of postnatal enteric neural stem cells. Stem Cells. 2021; 39: 1236– 1252. https://doi.org/10.1002/stem.3388.

**Fetoscopic fixation of the fetal membranes using new innovative techniques on an avital model**
ID: 377

**Kategorie: DGKCH - Kinderchirurgische Forschung**

Eva Ana Menke[1], Clint Hansen[2], Lidya-Olgu Durmaz[1], Helene Zerwer[1], Robert Bergholz[1], Andreas Meinzer[1]

[1]*UKSH, Klinik für Allgemeine-, Visceral-, Thorax-, Transplantations- und Kinderchirurgie, Kiel, Deutschland*, [2]*UKSH, Klinik für Neurologie , Kiel, Deutschland*

**Background:**
Despite significant advancements in perinatal care, certain congenital disorders continue to exhibit high rates of mortality and morbidity both in utero and during the postnatal period. Fetoscopic surgery has emerged as a crucial intervention for selected pathologies, potentially altering disease progression through early therapeutic measures. But the risks and complications associated with fetoscopic surgery, such as chorioamniotic membrane separation (CMS) or preterm premature rupture of membranes (PPROM)represent a significant disadvantage compared to open surgical procedures and could negate the advantages of minimally invasive interventions. Most established techniques to prevent CMS and PPROM are currently only applicable through open surgery, except for chorioamniotic plication as described by Belfort, but the latter still utilizing a laparotomy to gain access to the uterus. However, to utilize the advantage of a minimally invasive fetoscopic procedure, this study aims to test different techniques for membrane plication in an inanimate model.

**Materials and methods:**
The study was conducted in two parts. In the first part, membranes were constructed to simulate fetal amniotic and chorionic membranes. In the second step an artificial model was built with the established membranes to simulate the intrauterine chorioamniotic environment for testing membrane separation during different plication techniques, such as T-fasteners (n=121), spinal needle (n=18), the Belfort Technique (n=121) and conventional laparoscopic ports (n=15). Those were assessed on their impact on membrane separation and movement by an optical three-dimensional motion capture using 12 cameras and multiple precision sensors (Qualisys AB, Gothenburg, Sweden.

**Results:**
The results demonstrate a significant difference in simulated amniotic and chorion membrane maximum and mean movement using the T-anchor and the Belfort suture technique ($p<0.001$). However, no significant difference was found for the maximum separation of the membranes between the use of both techniques ($p>0.325$). The distance between the membranes appears to be critical in the pathogenesis of PPROM and CMS.

**Conclusion:**
Although significant differences were observed between the suture technique and the T-anchor regarding deviations for the individual membranes, there appears to be no difference in the invasiveness of membrane separation of the two techniques.

Abstracts – DCK 2025 – 142nd Congress of the German Society of Surgery – Munich, March 26–28 • DOI 10.1515/iss-2025-2004
Innov Surg Sci 2025; 10 (Special Suppl 1): s209–s255

### Investigating metabolic changes in Hirschsprung colon organoids under acute and subacute proinflammatory stress using the Seahorse Extracellular Flux Analyzer

ID: 493

**Kategorie: DGKCH - Kinderchirurgische Forschung**

Anna Romanova[1], Johanna Hagens[1], Pauline Schuppert[1], Zhongwen Li[1], Lucie Rohwäder[1], Hans Christian Schmidt[1], Laia Pagerols Raluy[1], Magdalena Trochimiuk[1], Konrad Reinshagen[1], Christian Tomuschat[1]

[1]*University Medical Center Hamburg-Eppendorf, Department of Pediatric Surgery, Hamburg, Deutschland*

**Background:**
This study aimed to develop and optimize a novel protocol for analyzing the bioenergetics of intestinal organoids using the Seahorse XFe96 Analyzer. We compared organoids derived from human ganglionic Hirschsprung disease and healthy control colon tissues, with a focus on metabolic changes in response to pro-inflammatory stimuli.

**Materials and methods:**
Mature intestinal organoids, cultivated from adult stem cells in colonic epithelium of both Hirschsprung and healthy subjects, were disrupted mechanically and enzymatically before being seeded onto Seahorse XFe96 plates. The organoids were exposed to a pro-inflammatory cocktail containing TNFα, IL-1β, and IL-6. Mitochondrial stress was induced using the Seahorse XFe96 Analyzer, and results were normalized to enable precise metabolic assessments under inflammatory conditions.

**Results:**
We successfully optimized the protocol, determining the ideal organoid quantity per well for accurate analysis. Initial findings show significant bioenergetic differences between Hirschsprung and control organoids, particularly under pro-inflammatory stress. Hirschsprung organoids exhibited higher baseline and stress-induced mitochondrial respiration, along with greater maximal respiratory capacity and proton leak compared to controls. These results suggest that Hirschsprung organoids have more active but less efficient mitochondrial networks under inflammatory conditions.

**Conclusion:**
This study addresses key technical challenges in organoid research and establishes a reliable method for analyzing 3D organoid bioenergetics. Our findings contribute to understanding the metabolic aspects of Hirschsprung's disease, especially in relation to inflammation, and provide a foundation for further applications of organoid technology in biomedical research.

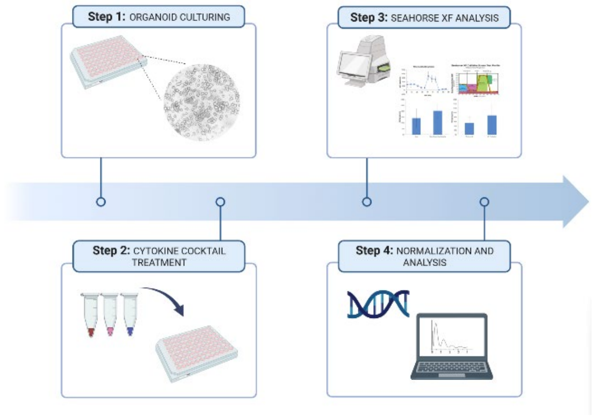

**Figure 1:** The diagram outlines a four-step experimental process used in cellular research. Step 1 involves culturing organoids, followed by Step 2, where the organoids are treated with TNAα, Il6 und IL-1β. In Step 3, cellular metabolism is analyzed using Seahorse XF technology, and finally, Step 4 involves data normalization and further analysis.

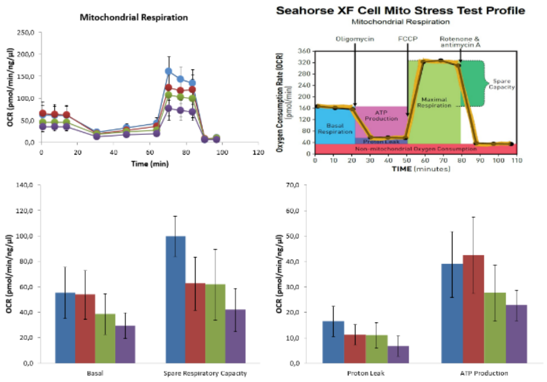

**Figure 2:** This figure presents the results of a Seahorse XF Cell Mito Stress Test performed on Hirschsprung organoids. The Oxygen Consumption Rate (OCR), measured in pmol/min/ng/µl, tracks mitochondrial respiration, with phases including basal respiration, ATP production, proton leak, and spare respiratory capacity. Key metabolic modulators, such as Oligomycin, FCCP, Rotenone, and Antimycin A, were introduced at specific time points to assess mitochondrial function under stress. The graph illustrates OCR changes over a 110-minute period, reflecting how cells from Hirschsprung organoids respond to mitochondrial stressors.

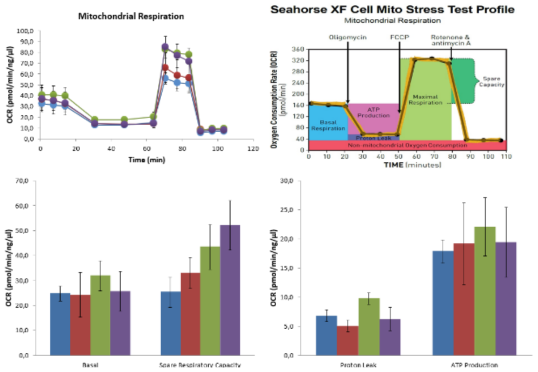

**Figure 3:** This figure shows the Oxygen Consumption Rate (OCR) data from a Seahorse XF Cell Mito Stress Test conducted on the control group. The test tracks mitochondrial respiration over time, starting with basal respiration, followed by ATP production, proton leak, and spare respiratory capacity. Mitochondrial modulators such as Oligomycin, FCCP, Rotenone, and Antimycin A were sequentially introduced to assess mitochondrial function.

### Pulmonary function testing in adolescent survivors of left-sided congenital diaphragmatic hernia and correlation of results to the shape of the reconstructed diaphragm

ID: 503

**Kategorie: DGKCH - Kinderchirurgische Forschung**

Christoph von Schrottenberg[1], Julia Elrod[2], Kaja Riebesell[2], Carolin Riemer[2], Jonathan Endlein[2], Richard Martel[2], Michael Boettcher[2], Kathrin Zahn[2]

[1]*Universitätsklinikum Carl Gustav Carus Dresden, Klinik und Poliklinik für Kinderchirurgie, Dresden, Deutschland*, [2]*Universitätsmedizin Mannheim, Klinik für Kinder- und Jugendchirurgie, Mannheim, Deutschland*

**Background:**
Congenital diaphragmatic hernia is a life-threatening condition in which infants are faced with lung hypoplasia, pulmonary hypertension and left heart dysfunction. After surgical reconstruction of the diaphragm and complex intensive care treatment with or without the need for neonatal extracorporeal membrane oxygenation (ECMO), patients are discharged. Long-term comorbidities in these patients include prolonged pulmonary hypertension and obstructive as well as restrictive pulmonary compromise making interdisciplinary long-term follow-up programs for these patients imperative. Within these programs it is essential to track lung function in order to offer the best medical support when needed.

**Materials and methods:**
26 adolescent survivors of left-sided CDH were subjected to pulmonary function testing within a standardized follow-up program at a tertiary referral center. Test results were converted into z-scores according to the European Respiratory Society Global Lung Function Initiative reference values. Patients were considered to have moderate to severe obstructive or restrictive pulmonary compromise when their z-scores for the respective pulmonary function test parameter was below -2.50. Pulmonary function test results were then correlated with previously defined radiological parameters in chest radiographs longitudinally assessed through early follow-up.

**Results:**
Average age at the time of pulmonary function testing was 13.9 years. Pulmonary function testing showed a reduced general lung function. Mean z-scores for forced expiratory volume in 1 second ($FEV_1$), forced vital capacity (FVC), $FEV_1$/FVC, forced expiratory flow between 25 – 75% of FVC ($FEF_{25/75}$), total lung capacity (TLC) and expiratory reserve volume (ERV) were all negative (-2.7 ± 1.5; -2.3 ± 1.7; -0.94 ± 1.4; -3.52 ± 2.0; -0.60 ± 1.0; -0.98 ± 1.2). Mean z-scores for functional residual capacity (FRC), residual volume (RV) and RV/TLC were all positive (0.39 ± 0.6; 1.0 ± 0.6; 1.84 ± 1.0). Patients with large diaphragmatic defects needing patch repair presented with significantly reduced FEV1 (-3.2 vs. -0.61, p <0.0001), FVC (-2.81 vs. -0.13, p = 0.0003), TLC (-0.85 vs. 0.41, p = 0.0059) and ERV (-1.25 vs. 0.12, p = 0.0221) compared to patients whose diaphragm was reconstructed primarily. No differences in pulmonary function testing were noted between patients with and without ECMO during the neonatal period. Moderate to severe obstructive pulmonary compromise was detected in 12 – 67% of patients depending on which parameter was assessed. Moderate to severe restrictive pulmonary compromise was detected in 0 – 46% of patients depending on which parameter was assessed.
Z-scores for FVC, $FEV_1$, TLC, RV/TLC and ERV could be significantly correlated with several geometric variables such as the left diaphragmatic diameter (LDD) or the left lower lung diameter (LLLD).

**Conclusion:**
Patients with CDH suffer obstructive and restrictive compromise of pulmonary function throughout adolescence, especially those with patch repair of the diaphragm. Reduced pulmonary function in adolescence correlates with some geometric variables of chest radiographs during early follow-up, hence these may serve as early prediction tools for long-term pulmonary compromise.

### 3D Organoids Derived from Extrahepatic Bile Ducts for Modeling Biliary Atresia
ID: 519

**Kategorie: DGKCH - Kinderchirurgische Forschung**

Zhongwen Li[1], Johanna Hagens[1], Lucie Rohwäder[1], Hans Christian Schmidt[1], Laia Pagerols Raluy[1], Magdalena Trochimiuk[1], Anna Romanova[1], Konrad Reinshagen[1], Christian Tomuschat[1]

[1]*University Medical Center Hamburg-Eppendorf, Department of Pediatric Surgery, Hamburg, Deutschland*

**Background:**
Biliary atresia is the most common congenital disorder affecting the extrahepatic biliary tract[1]. The intrahepatic and extrahepatic biliary systems develop independently, and the mechanisms by which they connect remain unclear. As a result, the development of the intrahepatic and extrahepatic biliary trees is typically discussed separately. Significant progress has been made in understanding the mechanisms that govern the differentiation of intrahepatic cholangiocytes from hepatoblasts, and increasingly detailed descriptions of intrahepatic duct morphogenesis have emerged. However, there are still many unresolved questions concerning the molecular mechanisms that regulate intrahepatic duct formation, and our understanding of the differentiation, morphogenesis, and patterning of the extrahepatic biliary system remains incomplete[2]. The objective of our study is to establish three-dimensional (3D) organoids derived from extrahepatic bile duct cholangiocytes in biliary atresia (BA) with the purpose of investigating BA pathophysiology.

**Materials and methods:**
We utilized two distinct 3D cell culture techniques to generate organoids from extrahepatic bile duct cholangiocytes, both for BA and control groups. Gallbladders from BA patients were sectioned into small fragments (<1 $mm^3$) and treated with EDTA to isolate epithelial cells, while epithelial cells from the control group were obtained via mechanical dissociation. All epithelial cells were cultured in a conditioned medium supplemented with epidermal growth factor (EGF), R-spondin, and a DKK-1 cocktail, which inhibits canonical Wnt signaling while promoting non-canonical Wnt signaling. This medium supports long-term, large-scale expansion of functional and genetically stable organoids[3, 4]. Immunofluorescence staining with antibodies against CK19, CK7, Sox9, GGT, and ALB was employed to characterize the resulting organoids[5]

**Results:**
Several notable differences were observed between the BA and control organoids. The growth rate of BA organoids was slower compared to those of the control group. Control organoids reached maturity within seven days, whereas BA organoids required an additional 1–2 days. Both groups displayed a spherical morphology; however, BA organoids exhibited a thicker wall structure(Fig.1). Reduced expression of CK19 and CK7 was detected in BA organoids, reflecting the characteristic epithelial cell loss associated with BA. Additionally, a decline in SOX9 expression was observed in BA organoids, indicating lower differentiation potential under identical culture conditions(Fig.2).

**Conclusion:**
The methodologies applied in this study successfully generate organoids directly from extrahepatic cholangiocytes, faithfully recapitulating their structural and functional properties. Consequently, this organoid system represents a valuable in vitro model for studying cholangiocyte injury and regeneration in the context of biliary atresia.
Fig. 1

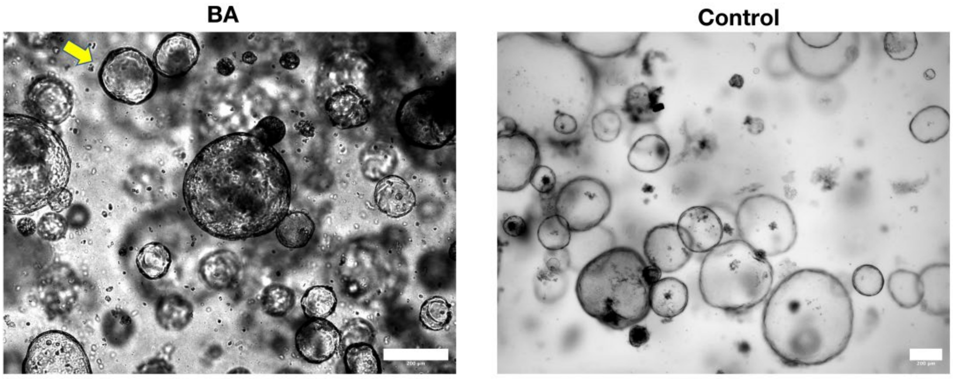

BA sample with a thicker wall structure.

Fig. 2

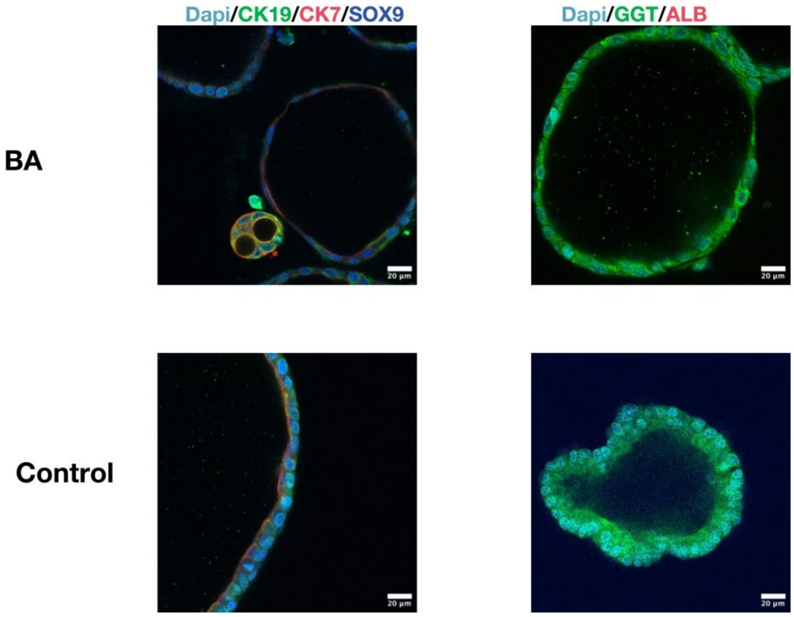

BA and control group IF stain. Both group shows ALB negative. CK19,CK7,SOX9 express lower in BA sample. In control group shows a great amount GGT lumen endocrine.

1. Lakshminarayanan, B. and M. Davenport, Biliary atresia: A comprehensive review. Journal of Autoimmunity, 2016. 73: p. 1-9.
2. Lemaigre, F.P., Development of the Intrahepatic and Extrahepatic Biliary Tract: A Framework for Understanding Congenital Diseases. Annu Rev Pathol, 2020. 15: p. 1-22.
3. Huch, M., et al., Long-Term Culture of Genome-Stable Bipotent Stem Cells from Adult Human Liver. Cell, 2015. 160(1): p. 299-312.
4. Huch, M., et al., Unlimited in vitro expansion of adult bi-potent pancreas progenitors through the Lgr5/R-spondin axis. The EMBO Journal, 2013. 32(20): p. 2708-2721-2721.
5. Tysoe, O.C., et al., Isolation and propagation of primary human cholangiocyte organoids for the generation of bioengineered biliary tissue. Nat Protoc, 2019. 14(6): p. 1884-1925.

**Prefrontal cerebral near-infrared spectroscopy for postoperative pain monitoring in infants**
ID: 543

**Kategorie: DGKCH - Kinderchirurgische Forschung**

Alaa Badawi[1], Ralf-Bodo Tröbs[2], Jochen Hubertus[1], Matthias Nissen[1]

[1]*Kindermedizinisches Zentrum der St. Elisabeth Gruppe - Klinik für Kinderchirurgie, Witten, Deutschland*, [2]*Krankenhaus Barmherzige Brüder Regensburg Klinik für Kinderchirurgie, Regensburg, Deutschland*

**Background:**
Detection of pain in infants is challenging given the nonverbal nature of pain responding in this age class. Pain detection is of importance concerning associated short- and long-term sequelae. According to literature, cortical processing of noxious stimuli in infants may be reflected by cerebral hemodynamics as measured by contralateral somatosensory cortex near-infrared spectroscopy (NIRS) signal. In this pilot study, we tried feasibility of measuring prefrontal cortical hemodynamic responses secondary to noxious stimuli by placement of NIRS optodes at the forehead which is devoid of hair in infants following elective surgery.

**Materials and methods:**
Prospectively collected data from healthy infants undergoing nociceptive heel lance/ squeeze (HLS) events following elective inguinal hernia repair at a tertiary Pediatric Surgery Department. Continuous prefrontal cerebral NIRS monitoring (c-r$SO_2$), adjusted to sidedness of nociceptive stimulus (ipsi- vs. contralateral), was performed in addition to physiologic (heart rate, breathing frequency, $SpO_2$) and behavioral (Children's and Infants Postoperative Pain Score; CHIPPS) pain assessment. Averages were calculated over 2 minutes epochs before (T1), during (T2) and after (T3) a HLS event, which was of variable duration. The primary outcome was the correlation between cerebral NIRS and CHIPPS. One-way repeated measure analysis of variance (ANOVA) or Kruskal–Wallis ANOVA were applied as appropriate in addition to repeated measurements correlation analysis.

**Results:**
Eight male infants received each one HLS event for routine blood gas sampling 3-6 hours after surgery when the caudal block had subsided. Mean (±SD) gestational age was 39 ± 2 weeks, age at surgery was 55 ± 21 days and weight was 5.2 ± 0.8 kg. We did not observe any correlation between cerebral NIRS and CHIPPS. Compared to baseline values, a transient increase in CHIPPS was elicited (p= 0.007) during HLS, but no changes were observed regarding ipsilateral and contralateral c-r$SO_2$, HR, BF, or $SpO_2$.

**Conclusion:**
In this study, only CHIPPS but not cerebral NIRS, HR, BF, and $SpO_2$ were altered upon nociceptive stimulus. Since we failed to directly measure prefrontal cortical hemodynamic responses to noxious stimulation, clinical use of frontal NIRS for pain assessment cannot be supported by our data. However, complementary use of somatosensory NIRS with behavioural measures such as CHIPPS may help optimizing analgesic therapy in this vulnerable cohort.

Abstracts – DCK 2025 – 142nd Congress of the German Society of Surgery – Munich, March 26–28 • DOI 10.1515/iss-2025-2004
Innov Surg Sci 2025; 10 (Special Suppl 1): s209–s255

**Patient-derived 3D cell models for neuroblastoma tumours - characterization and potential therapy testing.**
ID: 556

**Kategorie: DGKCH - Kinderchirurgische Forschung**

Melanie Le[1], Constantin Jennissen[1], Konrad Reinshagen[1], Christian Tomuschat[1], Laia Pagerols[1]

[1]*University Medical Center Hamburg-Eppendorf, Department of Pediatric Surgery, Hamburg, Deutschland*

**Background:**
The prognosis for patients with high-risk neuroblastoma remains poor despite intensive multimodal therapy, underscoring the need for personalized treatment approaches that allow for preclinical drug testing. Patient-derived neuroblastoma models, including 3D spheroids, are essential for evaluating drug responses. Spheroids have been proven to mimic the metabolic and proliferation gradients of the in vivo tumor. A variety of culture techniques have been introduced – scaffold-free in suspension or using gel-based scaffold materials like Matrigel or Cultrex, and it is believed that growing spheres in gel-like scaffolds might be better to preserve tumor characteristics.

**Materials and methods:**
Human primary neuroblastoma tumor samples (n=5) were obtained from pediatric patients undergoing surgery. Frozen tumor tissues were disaggregated, and single-cell suspensions were gel-embedded in Matrigel or Cultrex for at least 30 days. Immunofluorescence was performed for neuroblastoma markers GD2, PHOX2B, and PGP9.5. Furthermore, flow cytometry (FACS) was used to further characterize cell subtypes (NCAM/CD56, GD2-Marker, CD117 (c-kit) and CD133), including CD45 to exclude leukocytes.

**Results:**
Sphere formation in gel-scaffolding technique was successful in 60% of cultures (3/5) within 1-2 months, with a stable increase in viable cell numbers over the culture period. All tested spheres were positive for PGP9.5, confirming neural origin. Spheres were dissociated to obtain single cells after 30 days. Dissociating spheres into single cells resulted in reduced viability, with only 12.9% vital cells recovered. FACS analysis confirmed neuroblastoma origin with 52.7% CD45-/CD56+ cells and high positivity for GD2 (86%). Cultures in standard tissue-treated dishes did not result in sphere formation, highlighting the need for low-attachment conditions.

**Conclusion:**
Patient-derived neuroblastoma spheroid models are feasible and maintain tumor characteristics, even in post-chemotherapy samples. However, the success rate of sphere formation and cell viability upon dissociation suggest that further optimization of culture protocols is necessary to improve reproducibility and cell recovery. The next step includes the test of novel therapy approaches on spheres.

**Graphics:**
Legend: Sphere formation after one month of culture. Left: Optical microscope, 20x enlargement. Right: Immunofluorescence Imaging. Anti-GD2 (Texas Red), Anti-PHOX2B (FITC), Anti-PGP9.5 (Alexa Flour Plus 680).

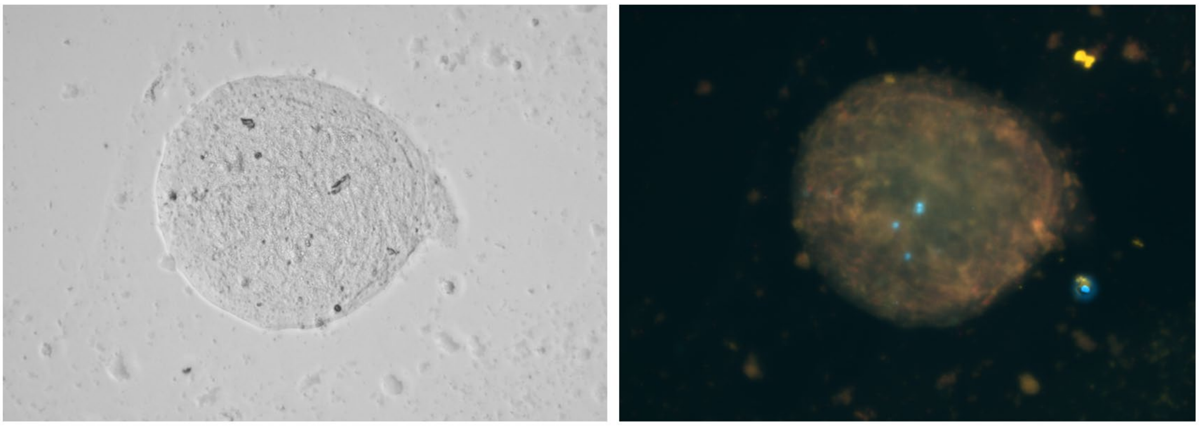

### Functional Outcome of Scapulectomy in Children – Single Center Experience and Systematic Review Of The Literature

ID: 584

**Kategorie: DGKCH - Kinderchirurgische Forschung**

Tobias Jhala[1], Maximilian Holweg[1], Markus Dietzel[1], Justus Lieber[1], Jörg Fuchs[1]

[1]*Universitätsklinikum Tübingen Abteilung für Kinderchirurgie mit Poliklinik, Tübingen, Deutschland*

**Background:**
Recent advances in reconstruction of the shoulder girdle and scapula had a significant impact on functional outcome in adults that underwent oncologic scapulectomy. In children and adolescents scapula tumors are rare. Moreover, the growing skeleton and high functional demands in this age group may hinder transferability of the promising results achieved in adults. This study aims to explore the functional outcome and different reconstructive options used in children undergoing (partial) scapulectomy.

**Materials and methods:**
A single center retrospective analysis of scapula tumors in children was performed. Furthermore, a systematic review, qualitative and quantitative synthesis was conducted to investigate the functional outcome of children and adolescents undergoing Malawer II or Malawer III resection. A systematic search was performed in the following of databases: Pubmed, EMBASE and Web of Science. Quality assessment tools were applied among included studies.

**Results:**
In total 3 patients were deemed eligible for the single center retrospective analysis. The three children (two boys, one girl, aged 4 – 11 years) all had Ewing Sarcoma of the scapula. Two patients underwent partial scapulectomy (Malawer II resection and Acromion resection) and had a better functional outcome than the one child that underwent total scapulectomy (Malawer III resection). Concerning the systematic review, of the 714 initial search results 17 studies were eligible for inclusion. In total 47 patients were extracted from the 17 studies. The analysis showed that patients that underwent Malawer III resection had a significant better functional outcome if a reconstructive surgery other than humeral suspension was performed. Patients that underwent gleonoid-preserving Malawer II resection showed similar results with or without reconstruction. No significant difference between different types of reconstruction methods other than humeral suspension was found.

**Conclusion:**
Children and adolescents undergoing Malawer III resection benefit from a reconstructive procedure other than humeral suspension. Reconstruction using either endoprosthesis or extracorporal irradiation and reimplantation provide similar functional outcome after Malawer III resection. In Malawer II resection reconstructive procedures do not influence functional outcome.

### Establishment of neuroblastoma 3D cell culture models
ID: 606

**Kategorie: DGKCH - Kinderchirurgische Forschung**

Constantin Jennissen[1], Melanie Le[1], Christian Tomuschat[1], Magdalena Trochimiuk[1], Konrad Reinshagen[1], Laia Pagerols Raluy[1]

[1]*Department of Pediatric Surgery, University Medical Center Hamburg-Eppendorf, Hamburg, Deutschland*

**Background:**
Traditional 2D neuroblastoma cultures have been shown to not fully recapitulate the heterogeneity and complexity of the original tumor tissue. Hence, 3D *in vitro* neuroblastoma models, in the form of spheroids or organoids, offer a new and more representative preclinical platform potentially able to provide more translatable data to the clinics. However, cultivation and development of patient-derived spheres with high reproducibility remain challenging and time-consuming. Furthermore, the lack of standardized protocols that guarantee high reproducibility make it difficult to culture reliable spheres. In this study, we aim to identify the crucial factors that influence sphere development and lifetime in order to generate a more standardized protocol with higher reproducibility, ultimately laying the foundation for drug response assays.

**Materials and methods:**
Six neuroblastoma stage 4 cell lines with and without MYCN-amplification were used to create neuroblastoma spheres in a systematically modified protocol. Based on preliminary tests four main cell culture parameters were tested for their potential effect on sphere development: 1) cell confluence as cell number per well, 2) cell culture medium, 3) type of the well-plate used and 4) the form of cultivation (embedded with matrix vs. not embedded).
Accordingly, 96-well plates were seeded with different cell numbers. Furthermore, sphere development was analyzed in different cell culture media (serum-free neuronal cell medium supplemented with several growth factors vs. RPMI Medium). Suspension and matrix-based cultures were compared. Matrix-embedded sphere-models were created by using either Matrigel or Cultrex as a scaffolding matrix. Suspension cells were additionally cultured by using different types of 96-well-plates. Spheres were manually quantified and qualified in terms of size and shape on day 3,5 and 7 after cultivation.
After selecting the optimal culture conditions, life expectation and viability of the spheres – especially important for future drug testing - were assessed by means of bioluminescent-based assays. OD values, which directly correlate with cell viability, were measured every 24 hours for 10 days.
Additionally, neuroblastoma spheres were characterized by means of immunofluorescence and microscopy to confirm the cell origin as neuroblastoma. For this purpose, GD2, PGP9.5 and PHOX2B were selected as neuroblastoma marker-set.

**Results:**
Our results demonstrate the influence of cell confluence and applied culture medium on sphere development. In the first case, seeding higher cell numbers seemed to be more favorable for sphere formation in suspension cultures, whereas lower cell numbers were better suited for the matrix-based cultures. Secondly, cultivation of Matrigel-embedded neuroblastoma cells with serum-free neuronal cell medium resulted in larger sphere size and higher sphere counts compared to the use of RPMI medium. Interestingly, successful sphere development cultured with RPMI was demonstrated in the suspension cultures. An additional influencing factor was the type and dilution of the scaffolding matrix. The use of Cultrex led to less frequent sphere formation compared to Matrigel. Regarding dilution of Matrigel, the undiluted and slightly diluted form tended to promote a higher sphere count compared to higher dilutions. Strikingly, our results suggest no advantages of our matrix-based cell model compared to suspension cultures in terms of size and shape of spheres.
Subsequently, first viability assays showed a peak at day 5 to 6 after cultivation for the cell lines tested. After that time point a steady decline in viability was observed, which was in accordance with their optical appearance.
Immunofluorescence-staining of neuroblastoma cells showed positive results for GD2 and PGP9.5, whereas staining of PHOX2B showed no clear results. Furthermore, no differences between matrix and suspension models were observed.

Abstracts – DCK 2025 – 142nd Congress of the German Society of Surgery – Munich, March 26–28 • DOI 10.1515/iss-2025-2004
Innov Surg Sci 2025; 10 (Special Suppl 1): s209–s255

**Conclusion:**
Although cultivation of the several cell lines is time-consuming and sphere development still appears to be heterogenic, also within the same cell line, our results demonstrate several conditions which promote sphere formation. These observations remark the importance of intensifying efforts to standardize a sphere-culture protocol, mandatory for intra- and inter-study reproducibility. The successful 3D-culture protocol in cell lines may be translated into the culture of neuroblastoma primary cells and thus support the establishment of spheres culture from patient material, enabling a more authentic representation of the original tumor. This, in turn, may promote the development of improved therapy strategies in a patient-specific manner.

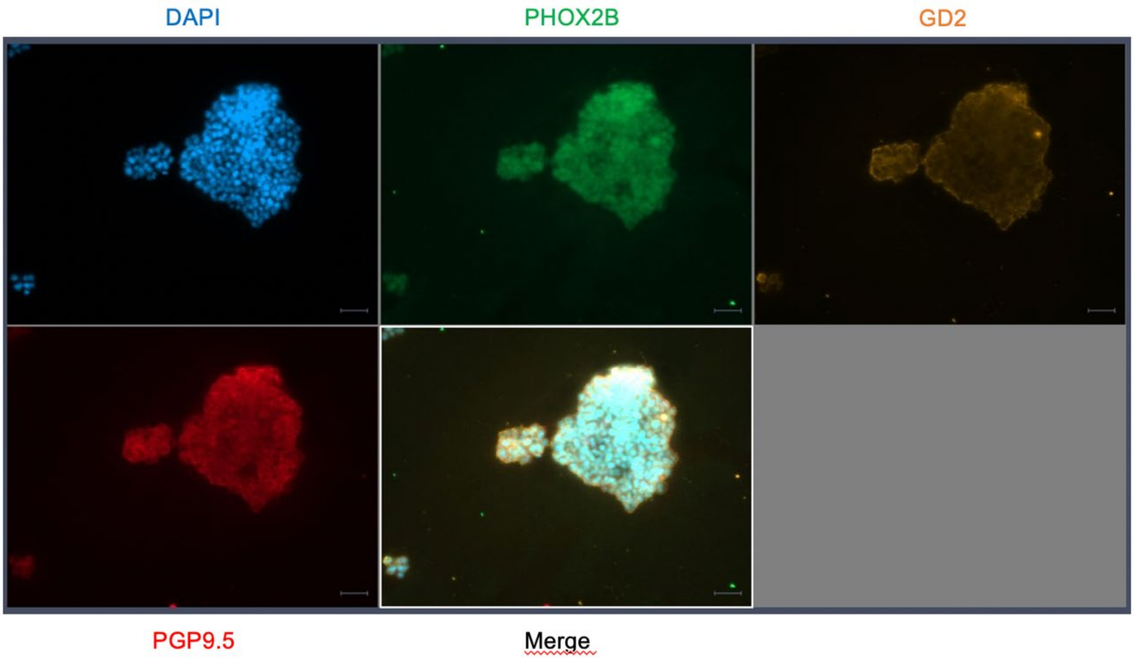

**Fig. 1** IF-staining of LAN-1 cells using the selected neuroblastoma marker set, day 5 after cultivation, 20x, scale bar 50µm

Abstracts – DCK 2025 – 142nd Congress of the German Society of Surgery – Munich, March 26–28 • DOI 10.1515/iss-2025-2004
Innov Surg Sci 2025; 10 (Special Suppl 1): s209–s255

### An Optical Tomography-Based Score to Assess Pediatric Hand Burns
ID: 754

**Kategorie: DGKCH - Kinderchirurgische Forschung**

Judith Lindert[1 2], Tina Straube[3], Beke Larsen[2], Julia Siebert[2], Eirini Liodaki[4], Kianusch Tafazzoli-Lari[2], Lutz Wünsch[2]

[1]*Rostock, Department of Paediatric Surgery, Rostock, Deutschland*, [2]*Lübeck, Department of Pediatric Surgery, University Hospital Lübeck, Lübeck, Deutschland*, [3]*Halle (Saale), Department of Paediatric Surgery, Halle (Saale), Deutschland*, [4]*Department of Plastic Surgery, University Hospital Lübeck, Schwerin*

**Background:**
Hand burns are among the most serious injuries in childhood and constitute 30% of the admitted burn injuries. Hand burns may have an enormous impact on a child’s future development. Our research aims to improve early burn wound assessments, allowing for early definitive wound care. Several imaging techniques are currently being investigated. Our study is based on optical coherence tomography (OCT), a technique that provides both morphological images of the skin and the blood flow to the papillary plexus of the skin.
We aim to define the morphologic pattern of pediatric hand burns as visualized via optical coherence tomography (OCT) and dynamic OCT (D-OCT).

**Materials and methods:**
We evaluated OCT scans of the palm and back of the hand to address three research questions:

1. Could optical coherence tomography be used to differentiate thermal injury from healthy skin in children with hand burns?
2. Is it possible to differentiate deep wounds eventually requiring skin grafts from superficial wounds that would heal spontaneously as early as 48 h after injury?
3. Can OCT patterns of hand burns be summarized into a clinically useful score?

Children admitted to the pediatric burn center at the University Medical Center Schleswig-Holstein - Campus Lübeck between 01/2016 and 12/2021 were eligible for this prospective longitudinal observational study. 67 hand burns in 48 children (0–15 years) were prospectively examined. Scans were interpreted by two independent observers. Relevant OCT findings were surface irregularity, loss of epidermis, loss of dermal pattern (skin lines or papillary spots, loss of surface regularity and irregular vascular pattern of the plexus papillaris. Score values were calculated retrospectively. dolor sit amet, consetetur sadipscing

**Results:**
To each feature, a value was assigned. Surface irregularity and epidermal loss each receive a maximum of one point. Dermal patterns and vascularity were scored from 0 to 2 . The score was 0 for healthy skin. The maximum score of 6 would indicate a deep burn with a destroyed dermal structure and absent dermal plexus.
A score of 4 was associated with spontaneous healing without the need for skin grafting, with a positive predictive value of 97%. Deeper wounds with delayed healing and/or the need of skin grafting received a score of 5 or above, with an agreement of medical healing in 80% and a positive predictive value of 56%.

**Conclusion:**
OCT and D-OCT reliably visualize the morphologic and microvascular patterns of pediatric burn injuries. The OCT images on the second day after injury show patterns that can be summarized in a clinical score.
The OCT burn score has the potential to support clinical decision-making and, subsequently, improve clinical outcomes and shorten hospital stays.

### Children with CDH are at high risk for severe pectus excavatum deformity and progressive thoracic asymmetry

ID: 815

**Kategorie: DGKCH - Kinderchirurgische Forschung**

Carolin Riemer[1], Luisa Gräfin Lambsdorff[1], Nina Hutflesz[1], Christoph Mohr[1], Meike Weis[2], Christel Weiss[3], Richard Martel[1], Michaela Klinke-Petrowsky[1], Katrin Zahn[1], Thomas Schaible[4], Michael Boettcher[1], Julia Elrod[1]

[1]*Universitätsklinikum Mannheim, Klinik für Kinder- und Jugendchirurgie, Mannheim, Deutschland*, [2]*Universitätsklinikum Mannheim, Klinik für Radiologie und Nuklearmedizin, Mannheim, Deutschland*, [3]*Medizinische Fakultät Mannheim, Medizinische Statistik, Biomathematik und Informationsverarbeitung, Mannheim, Deutschland*, [4]*Universitätsklinikum Mannheim, Klinik für Neonatologie, Mannheim, Deutschland*

**Background:**
Congenital diaphragmatic hernia (CDH) survivors exhibit an increased risk for developing musculoskeletal anomalies, which significantly contribute to their long-term disease morbidity. This prospective long-term cohort study investigates the characteristics, predictors and dynamic changes of different chest wall deformities in a large cohort of CDH patients.

**Materials and methods:**
All children diagnosed with CDH and treated at the Department of Pediatric Surgery at the University Hospital Mannheim, from 2010 to 2023, were included. Thoracic morphology was evaluated based on 740 CT or MRI scans. Measurements of the thoracic cavity were analyzed in relation to patient-specific variables, markers of disease severity and treatment-related factors. Statistical analysis was performed using the Mann-Whitney-U-Test and Fisher's Exact Test. P-values $< 0.05$ were considered statistically significant.

**Results:**
Out of 1,216 children with CDH treated at our hospital from 2010 to 2023, 548 received cross-sectional imaging examinations of the chest within the follow-up program. Mean Haller index was $2.62 \pm 0.55$ and pectus excavatum deformity (Haller Index $> 2.7$) was present in 34.09% of CDH patients. Thoracic asymmetry in favor of the right hemithorax (Asymmetry Index $< 0.95$) was present in 45.90% of patients with left-sided CDH. Thoracic asymmetry showed a pronounced age-related progression when comparing patients around 10 years old to those in the 2-year cohort, with a decrease of the mean Asymmetry Index from 0.96 to 0.94 ($p = 0.0046$) and a decrease of the mean Area-Ratio from 0.95 to 0.89 ($p < 0.0001$).
The severity of chest wall deformities showed a significant correlation with prenatal diagnosis, liver herniation, lower lung to head ratio and fetal relative lung volume, use of ECMO and inhaled nitric oxide, diaphragmatic patch closure and laparotomy.

**Conclusion:**
Children with CDH show an increased incidence of chest wall deformities, including pectus deformity and asymmetry with the potential for significant age-related progression. Measurement of thoracic morphology using standardized parameters based on MRI or CT scans should be implemented as an essential component of standardized follow-up programs. In addition, rigorous randomized, controlled intervention trials are required to evaluate whether certain therapies can alter the trajectory of these deformities, and to confirm whether the correlations with disease and treatment related factors are indeed causal in nature.

**SuSiPed 1.0 and 2.0 laparoscopic education start basic then go to advanced**
ID: 511

**Kategorie: DGKCH - MIC: Simulation in der minimal-invasiven Kinderchirurgie**

Andreas Lindner[1], Nora Weisser[1], Sophia Jahrstorfer[1], Julia Haehl[1], Jan Gödeke[1], Oliver Muensterer[1]

[1]*LMU University Hospital, Department of Pediatric Surgery, Munich, Deutschland*

**Background:**
As simulation-based training in minimal invasive surgery is gaining importance, efforts should focus on how to make it more efficient and time-effective.

**Materials and methods:**
We used the SuSiPed 1.0[1] and the SuSiPed 2.0[2] curriculum in a self-made minimal invasive box trainer and a 30degree optic camera. The (basic) SuSiPed 1.0 curriculum consists of 5 tasks (numbered Task1-5): shell-transfer, gauze cutting, cyst resection, simple interrupted suture and creation of a slipknot. The (advanced) SuSiPed 2.0 curriculum consists of 6 tasks (numbered Task 6-11): simple stitching, continuous stitching, 3D-knot-tying, general knot-tying, simulation of a fundoplication using slipknot technique, and esophageal anastomosis using slipknot.
Medical students with no prior laparoscopic training were recruited as study participants and divided into two groups: Group one started with SuSiPed 1.0, followed by the SuSiPed 2.0 curriculum. Group two performed the curriculum in reverse: they started with SuSiPed 2.0, followed by SuSiPed 1.0. Instructions for accomplishing the tasks were given by video tutorial prior to the hands-on training. The camera operator and instructor were always the same person. All trainees repeated each curriculum three times on a different day. Time to completion was measured as the primary outcome parameter. For SuSiPed 1.0 we measured only the second and third time, due to proband acquisition via workshop. The number of errors committed during completion of the modules were assessed as a secondary parameter.
For analysis, the mean time required to complete the tasks, as well as the mistakes made in each task were compared using the t-welch test ($p<0.05$ was considered significant and $p<0.10$ was considered a trend). Effect size was calculated using Cohen's d test.

**Results:**
Both groups improved with practice, indicated by higher scores in the second curriculum. Overall time to completion trended to be lower in group 1, which started with SuSiPed 1.0 (see table).
There were no significant differences in mistakes made.

| | Group1 | n=11 | Group2 | n=7 | | |
|---|---|---|---|---|---|---|
| | t in s | SD | t in s | SD | p | eff |
| Overall | 8167 | 1199 | 10027 | 2256 | 0.08 | **1.11** |
| Task1 | 792 | 192 | 730 | 209 | 0.54 | 0.31 |
| Task2 | 863 | 236 | 805 | 246 | 0.63 | 0.24 |
| Task3 | 370 | 217 | 364 | 202 | 0.95 | 0.03 |
| Task4 | 623 | 145 | 425 | 63 | **0.00** | **1.64** |
| Task5 | 696 | 134 | 537 | 143 | **0.04** | **1.16** |
| Task6 | 966 | 538 | 1680 | 512 | **0.00** | **1.35** |
| Task7 | 782 | 244 | 1265 | 485 | **0.04** | **1.36** |
| Task8 | 356 | 135 | 623 | 137 | **0.00** | **1.97** |
| Task9 | 765 | 228 | 1047 | 179 | **0.01** | **1.34** |
| Task10 | 1084 | 272 | 1405 | 430 | 0.11 | **0.94** |
| Task11 | 870 | 256 | 1145 | 374 | 0.12 | **0.90** |

| | Group1 n=11 | | Group2 n=7 | | | |
|---|---|---|---|---|---|---|
| | misstakes | SD | misstakes | SD | p | eff |
| Overall | 6.64 | 4.74 | 6.43 | 3.36 | 0.91 | 0.05 |
| Task1 | 1.55 | 1.75 | 2.00 | 2.16 | 0.65 | 0.24 |
| Task2 | 3.64 | 3.26 | 2.71 | 2.29 | 0.50 | 0.31 |
| Task3 | 0.09 | 0.30 | 0.29 | 0.49 | 0.36 | 0.52 |
| Task4 | 0.27 | 0.65 | 0.14 | 0.38 | 0.60 | 0.23 |
| Task5 | 0.27 | 0.47 | 0.14 | 0.38 | 0.53 | 0.30 |
| Task6 | 0.09 | 0.30 | 0.43 | 0.79 | 0.31 | 0.63 |
| Task7 | 0.09 | 0.30 | 0.14 | 0.38 | 0.77 | 0.15 |
| Task8 | 0.00 | 0.00 | 0.00 | 0.00 | N.a.N. | N.a.N |
| Task9 | 0.09 | 0.30 | 0.29 | 0.76 | 0.53 | 0.38 |
| Task10 | 0.45 | 0.69 | 0.14 | 0.38 | 0.24 | 0.52 |
| Task11 | 0.09 | 0.30 | 0.14 | 0.38 | 0.77 | 0.15 |

**Conclusion:**
Our study indicates that the sequence in which laparoscopic tasks are practiced during simulation training is essential. Overall performance showed a trend for improvement when the basic SuSiPed 1.0 course was taken before the advanced SuSiPed 2.0 course. This indicates that laparoscopic skills should be instructed in a sequence from simple to more complex tasks, and not in random order. A structured pedagogic program that initially teaches basic followed by more complex tasks is more effective, saves time, and skills may also decrease student frustration. More research like this is required to design the optimal laparoscopic training program.

References:
[1] Weisser N, Küppers J, Lindner A, Heinrich M, Zimmermann P, Muensterer OJ.
SuSiPed: An initial step towards a universal, low-cost, 3D-printable platform for pediatric minimal-invasive surgery training.
J Pediatr Surg 2023; 58(4): 675-678. https://doi.org/10.1016/j.jpedsurg.2022.12.018

[2] Julia Haehl, Sophia Jahrstorfer, Andreas Lindner, Jan Gödeke, Oliver Muensterer
SuSiPed 2.0: The next step towards a future practical examination for pediatric minimal-invasive surgery
Journal of Surgical Simulation (2024) 11, 35–42. https://doi.org/10.1102/2051-7726.2024.0004

**Complicated appendicitis with periappendicular abscess: Are laparoscopic appendectomies a froid superior to primary laparoscopic appendectomies?**
ID: 385

**Kategorie: DGKCH - Modernes Management der Appendizitis**

Benedikt Wilke[1], Ricarda Fischbach[1], Guido Seitz[1], Mohammad Vahdad[1]

[1]*Department of Pediatric Surgery and Urology, University Hospital Giessen-Marburg, Marburg, Deutschland*

**Background:**
Perforated appendicitis accounts for 20-30% of all cases of appendicitis (PA). Diagnosis is still challenging despite the use of ultrasonography as well as CT scan. The management of PA depends on the general condition of the patient, the nature of perforation and whether an abscess is present on imaging studies or not. Septic patients or patients with generalized peritonitis require preoperative resuscitation. The optimal treatment strategies including primary appendectomy or interval appendectomy following conservative antibiotic therapy remain controversial. In primary appendectomy, the patient undergoes surgery within 24 hours of diagnosis, during which any intra-abdominal abscess is drained or purulent fluid is removed. An appendectomy a froid is performed after a parenteral cool-down therapy with antibiotics with the anticipated advantage of the potential reduction of intra- and/or postoperative complications. A heterogeneity in success rates between these two approaches is reported in literature. In order to clarify these ambiguities, we evaluated the efficiency of interval appendectomy in cases of perforated appendicitis, in order to identify differences in treatment outcomes.

**Materials and methods:**
Patients undergoing appendectomy between 2016 and 2024 were retrospectively identified. We included 61 patients with sonographically (periappendicular abscess), intraoperatively macroscopically visible, or postoperatively pathologically confirmed perforations. Thirty-one patients were treated with an antibiotic cool-down therapy and subsequently underwent interval appendectomy. After resolution of the condition, patients were discharged, and interval appendectomy was performed four weeks later, accompanied by oral antibiotic therapy until surgery. Twenty-four patients underwent primary surgery. Six patients who did not respond to antibiotic therapy and required emergency appendectomy were excluded. Parameters assessed retrospectively included the length of hospital stay, duration of intravenous and oral antibiotic therapy, and operation time. Statistical analysis was conducted descriptively and by comparing means using the T-test.

**Results:**
Patients undergoing interval appendectomy were initially hospitalized for an average of 8.13 [± 3.07] days for cool-down therapy. This was followed by an outpatient oral antibiotic therapy for an average of 25.19 [± 9.51] days. The postoperative stay after appendectomy was significantly shorter for patients who underwent interval surgery [4.13 ± 1.7 vs. 7.25 ± 1.92; $p < .001$]. The average operation time showed no significant differences, with a mean increase of 1.33 minutes [$p = .852$] for primary appendectomies.

**Conclusion:**
Based on our data, there is no difference in outcome of interval appendectomy compared to primary surgery. Our data indicate a significantly shorter postoperative length of stay after secondary appendectomy. We hypothesize that antibiotic cool-down therapy leads to fewer perioperative complications and improved surgical conditions. At our center, a fellowship system is in place, allowing residents to regularly perform laparoscopic appendectomies under the guidance of a specialist. Consequently, the duration of surgery cannot be meaningfully assessed without considering the surgeon's level of training. In the future, this study will be supported by a qualitative survey-based study. An internal goal is to develop an algorithm for the standardized treatment of perforated appendicitis through further prospective data collection. Additional research, ideally within the framework of a multicenter study with large sample sizes, is necessary to establish evidence-based guidelines in Germany for perforated appendicitis in children. Finally, the economic dimension of treating this condition presents another area of research potential, particularly in comparing the cost-effectiveness and profitability of the two treatment approaches.

**Suspected appendicitis and acute abdominal pain in children and adolescents – a risk-based management demonstrating enemas to be diagnostic and therapeutic**
ID: 713

**Kategorie: DGKCH - Modernes Management der Appendizitis**

Benedikt Gross[1], Klaudia Lipinska[2], Arend Rahrisch[2], Julia Felber[2], Carolin Hellmund[2], Jurek Schultz[2], Guido Fitze[2]

[1]*Klinik und Poliklinik für Kinder- und Jugendmedizin am Uniklinikum Dresden, Dresden, Deutschland*, [2]*Universitätsklinikum Carl Gustav Carus, Klinik und Poliklinik für Kinderchirurgie, Dresden, Deutschland*

**Background:**
Many children and adolescents present to emergency departments with the suspicion of acute appendicitis or acute abdominal pain of varying causes. Many might be relieved by an enema-prompted bowel motion, but there is little data on enemas' potential risks. Since we lack standard risk assessments, many patients are subjected to potentially unnecessary diagnostics, while urgent therapies could be delayed for others.

**Materials and methods:**
We screened 7,831 emergency presentations for ICD diagnosis related to abdominal pain. We then analyzed 1,723 patients aged 3-17 years with acute abdominal pain as presenting complaints in the emergency department of our tertiary pediatric surgery between 2018 and 2019 for all clinical data, including symptoms, diagnoses, therapy, and clinical outcomes.

**Results:**
Abdominal pain is associated with 175 ICD codes and accounted for 7.4% of emergency presentations. 81% were pain-free and discharged after an enema, which reduced the rate of hospital admissions without later surgery while not increasing re-admissions or delayed operations. We recorded no complications after enemas. Among all tested symptoms and signs, only abdominal rigidity and pain migration typical for appendicitis were helpful in predicting the need for in-patient admission or surgery.

**Conclusion:**
We see no substantial risk in administering an enema to otherwise healthy children and adolescents with acute abdominal pain if there is no abdominal rigidity or typical migration of pain. No further diagnostic work-up is immediately necessary when patients are free of complaints after passing an enema-prompted bowel motion. This management can help spare the patients from unnecessary diagnostics and preserve health system resources.

**Non-surgical akute abdomen suspicious for appendicitis in childhood and adolecence. A retrospective monoinstitutional cohort study**
ID: 867

**Kategorie: DGKCH - Modernes Management der Appendizitis**

Ralf-Bodo Tröbs[1], Michael Peter Mayer[2], Tomascz Baranski[3], Matthias Nissen[4]

[1]*Paderborn, St. Vinzenz Kliniken , Klinik für Allgemein-, Viszeral- und Kinderchirurgie, Kinderchirurgie, Paderborn, Deutschland*, [2]*St. Vinzenz Kliniken, Klinik für Allgemein-, Viszeral- und Kinderchirurgie, Paderborn, Deutschland*, [3]*Helios St. Johannes Klinikum, Klinik für Kinderchirurgie, Duisburg, Deutschland*, [4]*Klinik für Kinderchirurgie, Marien Hospital Witten, St. Elisabeth-Gruppe, Witten, Deutschland*

**Background:**
The management of children with acute appendicitis has undergone a paradigm shift in the last decade. This is based on the realization that patients with low-grade, uncomplicated appendicitis can remain under a conservative care.

**Materials and methods:**
The data were originally collected retrospectively on 352 children and adolescents. The inclusion criteria were a suspected diagnosis of appendicitis, hospitalization and an observational approach versus surgery. Data from 17 patients were excluded. A total of 335 children remained in the study. Group 1 included all patients who did not undergo appendectomy, non-surgical group (n = 220). Group 2 consisted of children who had undergone laparoscopic appendectomy and in whom histological examination of the appendix had revealed only minimal findings (usually “catarrhal”). All patients had an initial ultrasound examination. The patients received continuous intravenous fluids and were initially fasting. The abdominal findings were checked regularly and ultrasound scans were repeated if necessary.

**Results:**
There was a female predominance. No significant differences were observed between the two groups with regard to age (10 years), admission temperature (p = 0.63), leukocyte count (p = 0.18), neutrophil-lymphocyte ratio (p = 0.88), or CRP value (p = 0.52). Similarly, no statistically significant differences were noted in Hb value (13.0 vs. 13.1 g/dl, p = 0.50) between the two groups. The median length of stay for 'non-surgical' was 2 days (1 to 6) and for 'surgical' was 3 days (2 to 7). Subsequently, bacterial enteritis pathogens were found in 23 cases, viral pathogens in 20 cases and parasites in 3 cases.

**Conclusion:**
In **conclusion**, the results show that children and adolescents in the non-surgical and surgical appendectomy groups come from a similar population. Retrospectively, they are candidates for conservative management. Depending on the findings, watch and wait with or without antibiotic therapy is risk-adapted for these patients and unnecessary interventions as well as LOS could be reduced.

### A proportional meta-analysis of lymph node involvement in pediatric patients and young adults with soft tissue sarcomas

ID: 198

**Kategorie: DGKCH - Onkologie: Schwerpunkt Keimzelltumoren**

Illya Martynov[1], Luca Tobi[2], Guido Seitz[1]

[1]*Zentrum für Kinderchirurgie des Universitätsklinikums Gießen-Marburg, Klinik für Kinderchirurgie und Kinderurologie, Marburg, Deutschland*, [2]*Zentrum für Kinderchirurgie des Universitätsklinikums Gießen-Marburg, Klinik für Kinderchirurgie, Gießen, Deutschland*

**Background:**
Soft tissue sarcomas (STS) account for 7% of pediatric cancers, with certain subtypes demonstrating a propensity for lymphatic spread. However, the variability in lymph node (LN) involvement rates reported in the literature makes it difficult to obtain a comprehensive overview. Our aim was to summarize existing data on LN involvement and identify high-risk patient groups by evaluating LN status across different histological subtypes and tumor locations.

**Materials and methods:**
We conducted a systematic literature search across PubMed, Scopus, and Web of Science. Of the 3,695 records initially identified, 180 articles were included in the meta-analysis. The inclusion criteria required that the articles report LN disease rates confirmed clinically, through imaging, or histologically. Only English-language articles with full-text availability were considered, and they had to include data on a minimum of three cases involving pediatric patients or young adults (0–21 years) with either rhabdomyosarcoma (RMS) or non-RMS soft tissue sarcomas (NRSTS).

**Results:**
Among the 33,579 RMS patients included in the analysis, the LN positivity rate was 24% [95% CI: 0.21–0.28]. The highest rates were observed in patients with alveolar RMS (42%), followed by undifferentiated RMS (28%) and mixed RMS (28%) histological subtypes, as well as in those with perianal/perineal (52%), extremity (35%), and head/neck (31%) tumor locations. Among the 7,052 NRSTS patients, the LN positivity rate was 9% [95% CI: 0.07–0.11], with desmoplastic small round cell tumor (41%), malignant rhabdoid tumor (22%), clear cell sarcoma (19%), and angiosarcoma (19%) showing the highest rates. Abdominal/pelvic (19%), gastrointestinal (14%), and thoracic (13%) tumor localizations were also associated with higher LN positivity.

**Conclusion:**
This meta-analysis of proportions summarizes the evidence of LN involvement across various studies in both RMS and NRSTS patients, encompassing different histological subtypes and tumor locations. The findings may assist clinicians and researchers in identifying high-risk groups and optimizing diagnostic and therapeutic strategies.

### Transient 21-Hydroxylase Deficiency due to Leydig Cell Tumour Mimicking Testicular Adrenal Rest Tumour: A Case Report and Literature Review

ID: 609

**Kategorie: DGKCH - Onkologie: Schwerpunkt Keimzelltumoren**

Katharina Maria Högg[1], Stefan Wudy[2], Hande Rakicioglu[2], Lars Berthold[3], Stefan Gattenlöhner[4], Marius Rohde[5], Jens Riedel[1], Illya Martynov[1], Guido Seitz[1]

[1]*Zentrum für Kinderchirurgie des Universitätsklinikums Gießen-Marburg, Klinik für Kinderchirurgie, Gießen, Deutschland*, [2]*Zentrum für Kinderheilkunde und Jugendmedizin , Klinik für Allgemeine Pädiatrie und Neonatologie , Gießen, Deutschland*, [3]*Klinik für diagnostische und interventionelle Radiologie und Kinderradiologie, Sektion Kinderradiologie, Gießen, Deutschland*, [4]*Universitätsklinikum Gießen-Marburg, Institut für Pathologie , Gießen, Deutschland*, [5]*Zentrum für Kinderheilkunde und Jugendmedizin , Klinik für pädiatrische Hämatologie und Onkologie, Gießen, Deutschland*

**Background:**
Testicular tumors are rare in children, accounting for 2–4% of all childhood cancers. They are classified into germ cell tumors and non-germ cell tumors, such as Leydig cell tumors (LCT). LCTs are characterized by their hormone-producing nature, primarily presenting in children with precocious puberty and painless swelling of the testis. An important differential diagnosis for LCT is the so-called testicular adrenal rest tumors (TART), which can arise due to congenital adrenal hyperplasia (CAH), a group of inherited autosomal recessive disorders characterized by enzyme defects in the steroidogenic pathways that lead to the biosynthesis of cortisol, aldosterone, and androgens. In contrast to LCT, which typically exhibits unilateral growth and can become malignant in up to 10% of cases, TARTs generally show bilateral growth without any tendency for malignancy. Differentiating between LCT and TART is of paramount importance as it has significant therapeutic consequences. While radical inguinal orchidectomy is the classical treatment for LCT, TARTs are managed with glucocorticoid therapy without the need for surgery.

**Materials and methods:**
Herein, along with a literature review, we present a rare case of LCT with transient 21-hydroxylase deficiency in a 5-year-old boy with precocious puberty, unilateral painless testicular swelling, and 21-hydroxylase deficiency.

**Results:**
Due to the rapid growth of the tumor, we performed a radical inguinal orchidectomy, with histological confirmation of LCT. The 21-hydroxylase deficiency normalized during follow-up.

**Conclusion:**
Our case report highlights the difficulty in distinguishing between LCT and TARTs, which lies in their overlapping symptoms and similar endocrinological findings.

**Machine learning (ML) solutions in general pediatric surgery: Which topics have already been developed recently?**
ID: 269

**Kategorie: DGKCH - Robotik in der Kinderchirurgie**

Holger Till[1], Sebastian Tschauner[2], Georg Singer[1], Tristan Till[3]

[1]*Medical University of Graz, Austria, Department of Paediatric and adolescent Surgery, Graz, Österreich*, [2]*Medical University of Graz, Department of Paediatric Radiology, Graz, Österreich*, [3]*Nanyang Technological University, Singapore, College of Computing and Data Science (CCDS). , singapore, Singapore*

**Background:**
The integration of artificial intelligence (AI) and machine learning (ML) into healthcare has significant potential to improve diagnostic accuracy and support clinical decision-making. However, the application of ML algorithms in pediatric surgery seems rather slow. Major reasons could be limited AI competencies on how to develop these algorithms and limited datasets in rare diseases. To become aware of AI solutions that have recently emerged in our field, we conducted a literature review analyzing recent topics and relevant models.

**Materials and methods:**
A comprehensive literature review was performed using PubMed and ChatGPT to identify articles that introduced newly developed ML solutions in general pediatric surgery, published between 2020 and 2024. We excluded review articles, meta-analyses, editorials, and case reports. The development process of each model was assessed according to Harvard Medical School (HMS) curriculum for AI in healthcare, which includes the following phases: 1. Conceptualization, 2. Data processing, 3. Model building and evaluation, 4. Explainability and integration into existing healthcare systems, and 5. Compliance and ethical considerations.

**Results:**
Our initial search yielded 331 articles, of which 64 were selected for full-text analysis. We excluded 50 publications related to other surgical specialties: cardiac surgery (n=14), neurosurgery (n=13), urology (n=9), orthopedics (n=7), surgical oncology (n=2), and others (n=5). Ultimately, we included 14 articles that specifically focused on general pediatric surgery. The topics of interest included appendicitis (n=5), Hirschsprung disease (n=3), necrotizing enterocolitis (n=2), intestinal perforation, cryptorchidism, and other conditions. All publications described the underlying data processing methods. ML algorithms employed included more than 10 distinct models, such as random forest, logistic regression, and decision trees. Model performance was evaluated using a range of metrics, including accuracy, precision, recall, and F1-score. Notably, none of the studies reported successful integration of their models into existing healthcare systems; however, all studies emphasized the necessity of ethical frameworks and regulatory compliance to foster trust and uphold patient rights

**Conclusion:**
Although newly developed AI/ML models in general pediatric surgery are relatively limited, they reflect advanced knowledge and technical competencies in ML. It remains imperative for pediatric surgeons to engage in the development of AI/ML technologies, as their expertise will help targeting patient oriented benefits and open doors for deployment in the healthcare system. To enhance the availability of larger datasets, particularly for rare diseases, our specialty should focus on building collaborative networks and improving data collection efforts.

### The Asensus Paediatric Registry of Robotic assisted Endoscopic Procedures in Urology, Gynaecology, and General Surgery (Asensus-Mini-Trust)

ID: 294

**Kategorie: DGKCH - Robotik in der Kinderchirurgie**

Jan Gödeke[1], Oliver Muensterer[1], Wim van Gemert[2], Ruben Visschers[2], Rianne Killaars[2]

[1]*Department of Pediatric Surgery, Dr. von Hauner Children's Hospital, LMU Munich, Munich, Deutschland*, [2]*Department of Pediatric Surgery, Maastricht University Medical Center +, Maastricht, Niederlande*

**Background:**
Robot assistance in the operating room is one of the important fields of development in pediatric surgery. In contrast to adult surgery, pediatric surgery is still in its early stages. This is partly due to the fact that there are currently only few manufacturers available, who address the surgical needs of children, especially toddlers and infants.
The Senhance system from Asensus is currently the only available robot assistance system that offers instruments with a diameter of 3 mm and 5 mm. Therefore it most closely matches the daily life reality of most minimally invasive pediatric surgeons.
In order to collect future data on pediatric surgical applications, performance and safety of the Senhance system with a higher number of cases, the Asensus-Mini-Trust Register was founded in October 2024. The two founding centers are located at the LMU Munich (DE) and the University of Maastricht (NL).

**Materials and methods:**
In both centers, anonymous case data on each robotically assisted operation is collected based on the specified criteria of a certified medical device and stored in a registry database. The requirements of DIN EN ISO 14155:2021-05 for clinical trials with medical devices are met.

**Results:**
The new register will be presented to the public for the first time as part of the DCK 2025. The first basic data from the founding members will also be shown.

**Conclusion:**
Although robotic assistance in pediatric surgery in 2025 is fundamentally feasible and safe in the hands of experienced surgeons, many questions remain unanswered, partly due to a lack of data registers. This weak point is now being addressed internationally. In the future, further international pediatric surgery centers will be gradually included in the Asensus-Mini-Trust Register, which will continuously increase the available data volume.

### Artificial intelligence (AI) based quality assurance algorithm for laparoscopic NISSEN fundoplication: An experimental proof of concept

ID: 310

**Kategorie: DGKCH - Robotik in der Kinderchirurgie**

Holger Till[1] [1], Ciro Esposito[2], Chung Kwong Yeung[3], Dariusz Patkowski[4], Sameh Shehata[5], Steve Rothenberg[6], Georg Singer[1], Tristan Till[7]

[1]*Medical University of Graz, Austria, Department of Paediatric and Adolescent Surgery, Graz, Österreich*, [2]*Federico II University of Naples, Italy , Pediatric Surgery Unit, Naples, Italien*, [3]*Chinese University of Hong Kong Shenzhen, Hong Kong, Paediatric Surgery, Hong Kong, Hong Kong*, [4]*Wroclaw Medical University, Wroclaw, Poland, Pediatric Surgery, wroclaw, Polen*, [5]*Alexandria Faculty of Medicine, Alexandria, Egypt, Pediatric Surgery, Alexandria, Egypt*, [6]*Rocky Mountain Hospital for Children, Denver, USA, Pediatric Surgery, Denver*, [7]*Nanyang Technological University, Singapore, College of Computing and Data Science (CCDS), Singapore, Singapore*

**Background:**
The adoption of artificial intelligence (AI) and computer vision (CV) for surgical quality assurance during major videoscopic procedures has been slow. One contributing factor may be the absence of predefined visual quality indicators (VQIs) specifically for each procedure that promise enough impact to improve surgical outcomes and enhance patient benefits. This study introduces the first AI/CV algorithm developed specifically for laparoscopic Nissen fundoplication, utilizing a set of VQIs to differentiate between correct and incorrect Nissen wraps

**Materials and methods:**
• Concept: A panel of six international experts from prominent academic institutions identified five key VQIs for an incorrect Nissen wrap: incomplete, twisted, too long (more than four knots), too loose, and positioned at/below the gastroesophageal (GE) junction.
• Data Collection: In a porcine model, three pediatric surgical residents and three consultants performed multiple iterations of both correct and incorrect Nissen fundoplications. Each procedure was recorded in short video segments from various angles and distances, with footage captured at 30 frames per second to ensure comprehensive visual data.
• Model Development: A total of 57 video sequences were processed, and images were extracted at intervals of 0.5 seconds, resulting in 3,138 relevant images after removing non-informative frames. These images were annotated for the presence of VQIs and divided into training (70%), validation (20%), and testing (10%) sets. The EfficientNet architecture was employed for image classification, optimizing the model to distinguish between correct and incorrect Nissen fundoplications based on the annotated VQIs.

**Results:**
The AI/CV models exhibited strong performance in predicting correct versus incorrect Nissen fundoplications based on the selected VQIs. Individual image classifiers reported an average F1-Score of 98.736% ± 1.01% when adjusted for optimal EER as decision boundary. Using a voting-based system, the individual detectors outperformed a single multi-class classifier, as well as a single binary classifier, when detecting incorrect versus correct Nissen.

**Conclusion:**
AI-driven surgical quality assurance algorithms represent a promising avenue for enhancing intraoperative decision-making and improving patient outcomes, particularly in videoscopic surgery. This study presents the first AI/CV model trained with visual quality indicators (VQI’s) to differentiate between correct and incorrect laparoscopic Nissen fundoplications. Our proof of concept establishes a foundation for further research into AI/CV applications for visual quality assurance in videoscopic surgery.
Ackowledgement: we thank Drs. B. Obermüller, A. Bokros, M. Eibisberger, C. Flucher, E. Friehs, P. Gasparella for their experimental support

**Figures:**

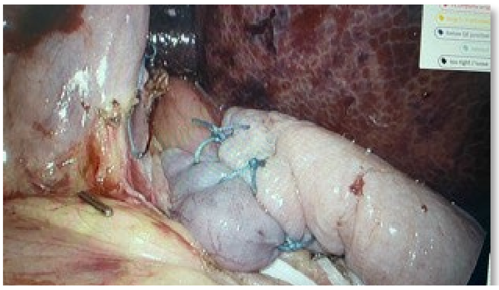

Figure 1: Complete, correct Nissen

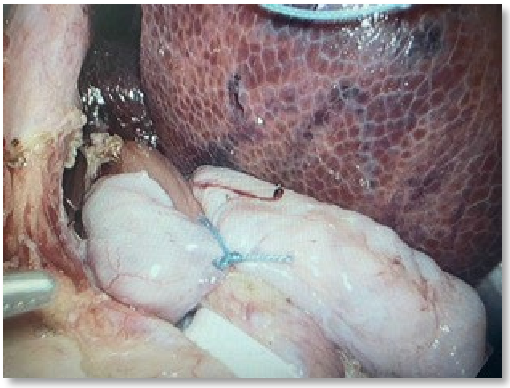

Figure 2: incomplete Nissen

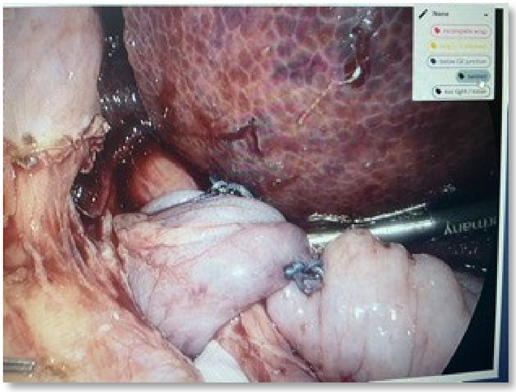

Figure 3: twisted wrap

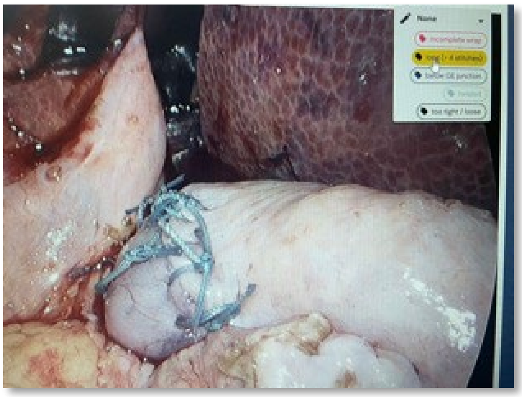

Figure 4: wrap is too long, at the GE junction

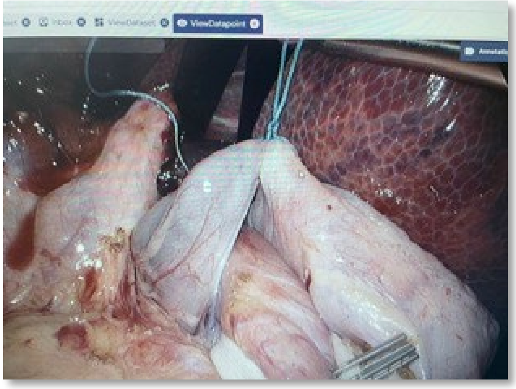

Figure 5: Loose wrap

**Pioneering Pediatric Robotic Surgery: Overcoming Challenges and Expanding Horizons in Minimally Invasive Techniques - report on 105 cases**
ID: 915

**Kategorie: DGKCH - Robotik in der Kinderchirurgie**

Martin Lacher[1], Gabriel Goetz[1], Richard Wagner[1], Richard Gnatzy[1], Jan-Hendrik Gosemann[1]

[1], *Universitätsklinikum Leipzig AöR, Klinik und Poliklinik für Kinderchirurgie, Leipzig, Deutschland*

**Background:**
Robotics has been successfully applied in adult surgeries, but adapting it for pediatric operations presents unique challenges due to the smaller anatomical dimensions of children. This study aims to evaluate the limitations and lessons learned from our pediatric robotic surgery program, initiated 3 years ago.

**Materials and methods:**
We evaluated patients who underwent robotic procedures with the da Vinci XI surgical system (Intuitive Surgical, Inc., Sunnyvale, CA) between October 2021 and September 2024. Parameters analyzed included surgical pathology, type of procedure, age, weight, conversion rate, operative time, and intra- and postoperative complications.

**Results:**
Between October 2021 and September 2024, 105 children underwent primary robotic-assisted laparoscopic surgery. Of these, 61 procedures (58%) were urological: pyeloplasty (N=24), robot-assisted laparoscopic extravesical ureteral reimplantation (RALUR; N=21), nephrectomy (N=5), heminephrectomy (N=5), and ovarian-sparing teratoma excision (N=6).

Fourty-four children (42%) had general surgical procedures: fundoplication/Heller myotomy (N=23), cholecystectomy (N=5), choledochal cyst excision with Roux-Y choledochojejunostomy (N=4), hemisplenectomy (N=6), abdominoperineal pull-through for Hirschsprung's disease (N=5) and ganglioneuroma (N=1).
Fifty-eight percent of all patients were female. The mean age at surgery was 4.9 years (range: 2.5 months–17 years) and median weight was 18,1 kg (range: 4.9 kg–73 kg). Mean console times were 89 minutes (range: 58–114 minutes) for pyeloplasty, 92 minutes (range: 60–115 minutes) for RALUR, and 55 minutes for fundoplication: (range 34-118). None of the cases required conversion to conventional laparoscopy, and no intraoperative complications occurred. One child, after RALUR, was readmitted due to transient bladder emptying issues, which resolved spontaneously within a month.
The main advantages of robotic surgery over conventional MIS in children include a stable magnified view, enhanced dexterity, improved surgeon ergonomics, and motion scaling that facilitates suturing in confined spaces.

**Conclusion:**
Our experience shows that technical and procedural skills from pediatric MIS can be effectively transferred to the robotic approach. At our center, the primary use is for reconstructive urological procedures, with no evident age or weight restrictions. However, challenges include the lack of haptic feedback, higher costs, and less favorable cosmetic outcomes due to the use of 8 mm rather than 3 mm trocars. Increased training is essential to shorten docking times. As robotic systems become more widely available, we expect this technology to play a pivotal role in the future of pediatric surgery.

**Congenital gastric duplication cyst: A case report of a 36 day-old girl**
ID: 338

**Kategorie: DGKCH - Safety Management / Komplikationsvermeidung in der Kinderchirurgie**

Simon Nwakor[1]

[1]*Universitätsklinikum Hamburg-Eppendorf, Kinderchirurgie, Hamburg, Deutschland*

**Background:**
As extremely rare congenital malformations, gastrointestinal tract duplications occur with a slight male predominance in about 1 of 4500 births [1]. In contrast to the more common cystic form, a tubular form can also be differentiated [2,3]. Gastric duplication cysts can appear with symptoms of gastric outlet obstruction, such as vomiting. As surgical therapy seems to be the only effective treatment, cyst excision has been established as preferred technique [4]. In order to share our experience with gastric duplication we present the case of a 36-day-old girl. The child presented with intermitting non-bilious vomiting. After initially quick weight gain, she showed signs of weight stagnation and an increased vomiting frequency after her first month.

**Materials and methods:**
We used a sonography of the abdomen to identify the gastric duplication and visualize the assumed gastric outlet obstruction. A primary laboratory chemistry enabled closer conclusions regarding the origin and the severity of the vomiting. A preoperative esophagogastroduodenoscopy provided further information about the cyst's location and its appearance. We decided to use an open laparotomy with gastrotomy as procedure for complete cyst excision and additionally performed a pyloromyotomy to treat pyloric hypertrophy. Remnants of the mucosal lining were carefully removed. In order to reduce postoperative infection, she received an antibiotic therapy for 7 days as well as antiacids and vitamin substitution. The increase in nutrition was gradual, initially by means of additional total parenteral nutrition.

**Results:**
In terms of laboratory chemistry, she remained stable with balanced electrolytes and unremarkable inflammation values. Using sonography, we identified a gastric duplication at the pyloric transition. We visualized a typical three-layered appearance and could display an intermitting obstruction of the gastric outlet, as well as a hypertrophic pyloric muscle. During esophagogastroduodenoscopy and the performed gastrotomy, an antric duplication could be localized 1cm proximal of the pyloric channel, in the dorso-lateral greater curvature. The 0,9 x 0,7 x 0,7 cm cystic mass impressed with a shared muscular wall but showed no signs of a deep communication to the gastric wall. After complete excision the postoperative course remained un-remarkable with no further vomiting. During the inpatient stay food intake could be switched successfully from parenteral nutrition to a free-form diet. Histopathological examinations confirmed the antric duplication cyst.

**Conclusion:**
Gastrointestinal duplication consists of a layer of smooth muscle, located within or adjacent to the gastrointestinal tract. The described mucous membrane lining is similar to the gastric segment but not adjacent [5,6]. With ultrasonography a hypoechogenic muscle as well as an inner echogenic mucosal layer can be distinguished [5,7], underlining the essential role of sonographic diagnostic in this case. In our opinion a primary surgical cyst resection remains essential. Agreeing with Arshad et al. 2018 it is necessary to reduce further complications for example malignant transformation [8]. We highly recommend complete cysts excision with dissection of the common wall between cyst and antrum. If the complete cyst excision is not possible, cauterization of the mucosa or mucosal stripping should prevent neoplastic changes and bleeding. Alternatively, the maintenance of a seromuscular gap as well as the use of a patch (e.g. omentum or colon) have been described so far [9]. We demonstrate that surgical treatment in cases of gastric duplication is mandatory. In the presence of pyloric hypertrophy, we recommend a combined cyst excision with pyloromyotomy.

References

1. Gupta, R. et al. Gastrointestinal Duplications: A Decade‘s Experience. Journal of Indian Association of Pediatric Surgeons 28, 128-136 (2023).
2. Ramakrishna, H.K. Intestinal duplication. The Indian journal of surgery 70, 270-273 (2008).
3. Rattan, K.N., Bansal, S. & Dhamija, A. Gastrointestinal Duplication Presenting as Neonatal Surgery 6,5 (2017).
4. Sharks, D., Bharany, R.P. & Mapshekhar, R.V. Duplication cyst of pyloric canal: a rare cause of pediatric gastric outlet obstruction: rare case report. The Indian journal of surgery 75, 322-325 (2013).
5. Gupta, S., Sleeman, D., Alsumait, B. & Abrams, L. Duplication cyst of the antrum: a case report.Canadian journal of surgery. Journal canadien de chirurgie 41, 248-250 (1998).
6. Hawkins, M.L., Lowery, C.H. & Mullen, J.T. Gastric duplication. Southern medical journal 67, 189 passim (1974).
7. Macpherson, R.I. Gastrointestinal tract duplications: clinical, pathogenic, etiologic, and radiologic considerations. RadioGraphics 13, 1063-1080 (1993).
8. Arshad, M., Jeelani, S.M., Siddiqui, A. & Rehan, R. Duplication cyst of the pylorous: a rare cause of gastric outlet obstruction. BMJ case reports 2018 (2018).
9. Lister, J., Irving, I.M. & Rickham, P.P., Ed’s. Neonatal surgery (Butterworths, London, 1990).

### Anesthesia and postoperative management of cytoreductive surgery (CRS) and hyperthermic intraperitoneal chemotherapy (HIPEC) in children– experience of a pediatric oncosurgical center

ID: 579

**Kategorie: DGKCH - Safety Management / Komplikationsvermeidung in der Kinderchirurgie**

Benedikt Richard Wagner[1], Sven Oberwegener[2], Cristian Urla[1], Andreas Schmidt[1], Martin Ebinger[2], Martin Eßlinger[2], Felix Neunhoeffer[2], Frank Fideler[3], Jörg Fuchs[1]

[1]*Universitätsklinikum Tübingen Abteilung für Kinderchirurgie mit Poliklinik, Tübingen, Deutschland*, [2]*Universitätsklinikum Tübingen - Klinik für Kinderheilkunde und Jugendmedizin, Tübingen, Deutschland*, [3]*Universitätsklinikum Tübingen, Anästhesiologie und Intensivmedizin, Tübingen, Deutschland*

**Background:**

Cytoreductive surgery (CRS) combined with hyperthermic intraperitoneal chemotherapy (HIPEC) has been shown to improve survival in pediatric patients with peritoneal sarcomatosis/carcinomatosis. Anesthetic management and pediatric intensive care can be challenging due to significant fluid, blood and protein losses, an increased metabolic rate, and nephrotoxicity, particularly during and after CRS with HIPEC.

**Materials and methods:**

We present the anesthetic management and intensive care strategies for patients undergoing CRS with HIPEC focusing on safety, intraoperative fluid and blood administration, pain control and management of procedure-associated adverse effects. Between January 2013 and august 2024, 27 patients (16 female, 11 male) underwent CRS and HIPEC at our institution. The indication for surgery was established by a multidisciplinary tumor board. HIPEC was performed using cisplatin (37.5-100mg/m2) and doxorubicin (15-30mg/m2) for 60 min. at 42°C. A retrospective review of patient's records was performed.

**Results:**

The most common diagnosis was rhabdomyosarcoma (n = 22). The median age at operation was 4 years [range 16 month to 20 years]. The median duration of surgery was 318 minutes [233 - 556]. The median intraoperative intravenous fluid intake was 86.2 ml/kg [28.8 - 147], and colloid administration was 23.8 ml/kg [0-53.7]. Blood loss was documented in 12 patients with a median loss of 7,6 ml/kg [1 - 48.6]. Fifteen patients received intraoperative transfusions, with a median red blood cell transfusion volume of 14.7 ml/kg [5.9 – 47.1]. Fourteen patients received intraoperative fresh frozen plasma transfusions (median 37 ml/kg, range 17.5 – 94.1). Mechanical ventilation was performed for a median duration of 27 hours [6.5 – 57.9]. No reintubation or additional respiratory support was necessary after extubation. Pleural effusions were documented by ultrasound in 16 patients before extubation, whereas in one patient pleural drainage had to be placed postoperative. The median urine output was 3.6 ml/kg/h [0.6 – 13.4] intraoperative, 4.1 ml/kg/h [1.3 – 8.2] on the first postoperative day, and 7.2 ml/kg/h [3.3 – 15.5] on the second day after surgery. Diuretics were necessary for 11 patients on the first postoperative day, and for 15 patients on the second day after surgery, with median dosing of 0.3 mg/kg/day [0.1 – 1]. No acute kidney injury was observed. The pain management was performed using two non-opiod analgesic and completed by a peridural catheter in all cases. The peridural catheter was removed after a median of 6 days [2–8]. Postoperative additional intravenous opioid was administered for a median of 2 days [1 – 11]. Nutrition could be started after a median of 3 days [1 – 15]. The median stay on the ICU was 2 days [1 – 6]. The patients could be discharged after a median of 11 days [8 – 20].

**Conclusion:**

CRS and HIPEC can be safely performed in children at centers of excellence in pediatric tumor surgery, staffed by experienced pediatric surgeons, anesthesiologists, and pediatric intensive care specialists. Renal protective strategies, such as calculated increased prerenal fluid intake, are essential to maintain adequate urine output and to avoid high doses of furosemide and cisplatin-associated nephrotoxicity. Effective postoperative pain management can be achieved through the use of epidural pain infusions, facilitating early nutritional support, gastrointestinal transit, and a shortened hospital stay.

**It is quality that counts rather than quantity - safety in pectus surgery**
ID: 739

**Kategorie: DGKCH - Safety Management / Komplikationsvermeidung in der Kinderchirurgie**

Frank-Martin Haecker[1], Thomas Krebs[1], Kai-Uwe Kleitsch[1]

[1]*Sankt Gallen, Ostschweizer Kinderspital, Klinik für Kinder- und Jugendchirurgie, St. Gallen, Schweiz*

**Background:**
For surgical repair of Pectus excavatum (PE), the Nuss procedure is considered today as the “gold standard” and will be performed with increasing frequency worldwide. In particular in centers of excellence, the method experienced numerous modifications such as routine thoracoscopy and/or sternal elevation, increasing safety of the procedure. Placement of multiple bars and/or the so called cross-bar technique were introduced to correct complex chest wall deformities. Standardized pain management, the introduction of cryoanalgesia and a standardized postoperative physiotherapy program including deep breathing exercises facilitate establishing an enhanced recovery after surgery (ERAS) process. Pectus surgeons practicing in small countries with low population are requested on the one hand to deliver excellent results comparable to high volume centers, but on the other hand to identify, adapt and introduce new modifications.

**Materials and methods:**
With a retrospective analysis we report on our experience with the introduction and routine use of intraoperative sternal elevation, application of the cross bar technique to correct complex deformities and the introduction of thoracal cryoablation as routine standardized pain management in our chest wall unit.

**Results:**
Within a 6.5 years’ period, a total of 61 PE patients (52 male, 9 female; age: 15.9 years; HI: 4.35) underwent surgical repair of PE. Due to pandemic related restrictions, we had 1 year with only 5 cases/year. Intraoperative sternal elevation using the vacuum bell was applied in every patient and improved safety of the procedure. In approx. 30%, placement of 2 bars was performed including the so called cross bar technique (50%) to correct complex and/or combined pectus deformities. With the introduction of cryoanalgesia, length of postoperative hospital stay decreased from 6.8 days to 3.3 days on average. Postoperative course was uneventful, major side effects were not observed.

**Conclusion:**
Also in small countries with low population, pectus surgeons may deliver excellent results comparable to high volume centers, even if they adapt and introduce new modifications. Summarizing our experience, we may conclude that it is quality that counts rather than quantity.

### Early removal of the abdominal patch is superior to late removal in children with Congenital Diaphragmatic Hernia

ID: 767

**Kategorie: DGKCH - Safety Management / Komplikationsvermeidung in der Kinderchirurgie**

Kaja Riebesell[1]

[1]*UMM Mannheim, Universität Heidelberg , Kinder- und Jungendchirurgie , Mannheim, Deutschland*

**Background:**
Congenital diaphragmatic hernia (CDH) presents a developmental anomaly of the diaphragm, resulting in displacement of intraabdominal organs into the thoracic cavity. It is associated with a spectrum of serious complications, including respiratory failure, pulmonary hypertension, pulmonary hypoplasia, gastrointestinal problems as well as orthopedic and neurological comorbidities and may reach life-threatening dimension. Clinical manifestations are highly variable and mortality ranges from 20% - 70%, with overall severity increasing with defect size. Surgical reconstruction of the diaphragm is often conducted after or during achieving cardiopulmonary stabilization through ECMO. In up to 70% of the patients, primary closure of the diaphragm can be achieved. In patients with large defect sizes, the abdominal cavity may be hypoplastic and abdominal wall extension using a patch is necessary to prevent abdominal compartment syndrome. However, data on predictors for the need of a patch and associated risks, such as infection or patch detachment, are limited.

**Materials and methods:**
We conducted a single-center, longitudinal, prospective cohort study to assess the outcome of neonates diagnosed with CDH who underwent surgical diaphragmatic reconstruction from 2000 to 2022 at the Department of Pediatric Surgery at the University Medical Center Mannheim, associated with the use of an abdominal patch. Patient-specific and disease-specific data from 1,000 neonatal CDH cases were analyzed, of which 152 (15.2%) required an abdominal wall patch (GORE-TEX® Soft Tissue Patch). The study assessed the prevalence of predictors necessitating the use of an abdominal wall patch, as well as the incidence of infection and complications.

**Results:**
A total of 152 required an abdominal wall patch. Predictive factors of the need to apply an abdominal wall patch were male sex, low birth weight, early gestational age, prenatal diagnosis of CDH, large defect size, liver-up. Patients with abdominal wall patches didn't show an elevated rate of ileus during follow-up, in comparison to children without abdominal wall patch. Of the 152 cases, a total of 94 patches (62.25%) were removed within the following years at a mean time of 340.0 days after primary surgery. Early patch removal (< 365 days), lead to a significantly shorter operation time (184.14 ± 48.46 min) than in the late removal group (216.37 ± 71.86 min; p = 0.0137). Complication rate was comparable in both groups (early n=18 (26.87%) vs late n=7 (25.93%), p = 0.7863) and early removal did not lead to an elevated rate of difficulties in closing the fascia. Most common reasons for patch removal were infections (n = 47(50.00%), elective removal (n =27(28.72%) and removal concomitantly during another surgery (n =13(13.83%). Regarding infected patches, the most common pathogen was Staphylococcus aureus with none of them being MRSA.

**Conclusion:**
This study evaluates subsequent surgeries and associated complications of abdominal patch placement in children with CDH. The results suggest that early patch removal is associated with a shorter surgery time and does not lead to an increased rate of complications. In cases of patch infection, we recommend empirical antibiotic therapy targeting staphylococcal bacteria in the absence of an antibiogram. Our findings aim to improve clinical decision-making and optimize outcomes for neonates with CDH.

**Lessons learned from longitudinal follow-up in neonates with and without ECMO-therapy allow for the implementation of preventive strategies to possibly reduce neurodevelopmental complications**
ID: 879

**Kategorie: DGKCH - Safety Management / Komplikationsvermeidung in der Kinderchirurgie**

Katrin Bettina Zahn[1], Thomas Schaible[1], Christel Weiss[1], Michael Boettcher[1], Rainer Kubiak[2]

[1]*Universitätsklinikum Mannheim, Mannheim, Deutschland*, [2]*Ostschweizer Kinderspital, Sankt Gallen, Schweiz*

**Background:**
In neonates with congenital diaphragmatic hernia (CDH), lung hypoplasia, pulmonary hypertension, a hypoplastic left ventricle and size of the diaphragmatic defect are a the main reasons for mortality and long-term morbidity. Extracorporeal membrane oxygenation (ECMO) is an invasive method for the treatment of pulmonary hypertension in these neonates. There is an ongoing discussion about its benefits and the possible risk of long-term neurological sequelae. This longitudinal cohort-study aims to identify the incidence and possible risk factors for neurodevelopmental delay after open CDH repair in neonates with and without ECMO-therapy to possibly implement preventive strategies.

**Materials and methods:**
Data were collected prospectively after ethical approval with standardized and validated neurological tests applied from six months to six years of age. All three different neurodevelopmental tests applied test for cognitive function as well as language and motor development. In order to be able to compare the results, these were classified from severe delay to development well above average according to standard deviation. 278 surviving ECMO- and nonECMO-patients were tested at different time-points during standardized long-term follow-up and a total of 784 neurodevelopmental tests were retrieved.
In order to possibly identify risk-factors for neurodevelopmental delay, univariable analysis between patients with and without retardation was performed for cognitive function (action strategies), expressive language and gross motor function as these were tested at all ages. Subsequently, all parameters which have been revealed as statistically significant have been considered for multiple logistic regression analysis, using the backward-selection method with a significance level of 0.05. the following parameters were tested: ECMO, fetoscopic endoluminal tracheal occlusion (FETO), CDH laterality, intrathoracic liver herniation in left-sided CDH, intrathoracic stomach herniation in left-sided CDH, defect-size, type of diaphragmatic reconstruction (primary versus patch), closure of the abdominal wall (primary versus patch), location of surgery (neonatal intensive care unit versus operating room), duration of surgery, duration of ventilation, duration of non-invasive ventilation, time to full enteral feeds, length of hospital stay, gender, birth weight, gestational age at birth, coexisting anomalies (in general, cardiac, pulmonary, abdominal), cerebral changes on the MRI at two years of age, weight at neurodevelopmental testing, and reoperation (in general and within the first year of life).

**Results:**
In 130 ECMO- and 148 nonECMO-patients, there was no significant difference regarding gender, gestational age and birth weight between groups. The ECMO-cohort included more severe CDH with significant differences concerning intraoperative findings (e.g. diaphragmatic patch: ECMO 99.2% vs. nonECMO 86.5%, p<0.0001) and postoperative treatment (e.g. invasive ventilation: ECMO 32 days vs. nonECMO 17 days, p<0.0001) as well as comorbidities during follow-up (e.g. re-operations: ECMO 50.8% vs. nonECMO 27%, p<0.0001). Cognitive deficits were observed significantly more often following ECMO at almost any age. There was no significant difference in language or motor development between ECMO- and non-ECMO patients. Long-term follow-up until school-age revealed a declining incidence of neurodevelopmental delay over time. Mainly, mild neurodevelopmental delay was detected and severe delay very rarily. In multiple regression analysis different independent risk-factors for cognitive delay at different time-points of follow-up were identified (e.g. at 1 year of age: ‚liver-up‘ 3.7 [1.2-11.4], p=0.0253; cardiac comorbidities 2.8 [1.2-6.4], p=0.0143). For delay in motor development weight, antenatal FETO and re-operation during the first year of life were detected as independent risk-factors at mutliple ages (e.g. at 1 year of age: weight (per kg) 0.6 [0.4-0.9], p=0.0071; FETO 18.3 [1.9-175.1], p=0.0116; re-operation within first year 4.5 [2.03-9.9], p=0.0002).

**Conclusion:**
Despite more severe CDH in ECMO-patients, mainly mild neurodevelopmental delay was observed during follow-up. Deficits of ECMO-patients regarding action strategies may rather reflect the severity of the disease itself than exposure to ECMO. The majority of ECMO- as well as non-ECMO-patients have a normal neurological outcome at school-age. Standardized follow-up with neurological testing helps to identify patients in need for special support early and prevent or reduce long-term problems. Malnutrition should be prevented, which also includes specific treatment of CDH-associated gastroesophageal reflux and constipation. Reoperations - especially during the first year of life - are an independent risk-factor at multiple time-points and for motor, language and cognitive development. Thus a meticulous technique during inital surgery as well as prevention of abdominal adhesions are important to reduce the need for secondary surgeries in these patients.

**Complications after pediatric appendectomy showed no significant variation based on surgeon experience, transection technique, or surgical approach**
ID: 943

**Kategorie: DGKCH - Safety Management / Komplikationsvermeidung in der Kinderchirurgie**

Nina Dietze[1], Michael Boettcher[1], Michaela Klinke Petrowsky[1], Patrick Thees[1], Deirdre Vincent[1]

[1]*Kinderchirurgie, Mannheim, Deutschland*

**Background:**
Appendectomy is one of the most common pediatric emergency surgical procedures, typically performed in cases of acute appendicitis. Despite its high success rates, postoperative complications can occasionally occur, potentially leading to extended hospital stays and increased morbidity. The risk of such complications, including intra-abdominal abscesses, wound infections, and other postoperative issues, is particularly elevated in cases of perforated appendicitis. This study aims to analyze the incidence and nature of complications arising during the same hospital admission following pediatric appendectomy. Furthermore, it seeks to assess whether variations in appendiceal transection techniques, the surgeon's level of experience, the stage of appendicitis, or the type and duration of antibiotic therapy influence postoperative outcomes

**Materials and methods:**
A retrospective analysis was conducted at our institution over the period from 2017 to 2024. The study population included pediatric patients who underwent appendectomy for acute appendicitis. Data collected encompassed demographic information, appendicitis severity (non-perforated vs. perforated), surgical approach (open vs. single-incision laparoscopic surgery [SILS] vs. conventional laparoscopy), technique of appendix transection (loop ligature vs. clip application vs. stapler), surgeon's experience level (consultant vs. resident), and antibiotic regimen (agent and duration). A propensity score matching was performed.
The primary outcome was the occurrence of postoperative complications necessitating reintervention during the same hospital stay, including wound infections, intra-abdominal abscesses, ileus, and other relevant postoperative conditions.

**Results:**
A total of 453 pediatric appendectomy cases were analyzed. Among these, 18 patients required reinterventions, with 17 of these cases involving initially perforated appendicitis. Of the 18 reinterventions, 14 involved abscess formation, 2 involved stump insufficiency, 1 involved an injury to the epigastric artery and 1 presented bowel adhesion. Reoperations were performed via relaparotomy, abscess-guided drainage, re-SILS, or computed tomography (CT)-guided drainage. All patients received postoperative antibiotic therapy.

**Conclusion:**
While appendectomy in children is generally a safe and effective procedure, complications, particularly in cases of perforated appendicitis, are not uncommon. No statistically significant differences were observed with regard to complication rates based on surgeon experience, appendiceal transection method, or surgical approach. Early diagnosis and precise management of complications remain critical for ensuring favorable outcomes. Further prospective studies are necessary to refine preventative strategies aimed at minimizing postoperative complications.
